# A pillar[5]arene-based planar chiral charge-transfer dye with enhanced circularly polarized luminescence and multiple responsive chiroptical changes†

**DOI:** 10.1039/d2sc06000k

**Published:** 2023-01-03

**Authors:** Jin-Fa Chen, Qing-Xiu Gao, Lijie Liu, Pangkuan Chen, Tai-Bao Wei

**Affiliations:** a Key Laboratory of Eco-Environment-Related Polymer Materials, Ministry of Education of China, Key Laboratory of Polymer Materials of Gansu Province, College of Chemistry and Chemical Engineering, Northwest Normal University Lanzhou Gansu 730070 P. R. China chenjinfa@nwnu.edu.cn +86 9317973191 +86 9317973191; b School of Chemistry and Chemical Engineering, Beijing Institute of Technology of China Beijing 102488 P. R. China; c College of Science, Henan Agricultural University Zhengzhou Henan 450002 P. R. China

## Abstract

The fabrication of circularly polarized luminescent (CPL) organic dyes based on macrocyclic architecture has become an importantly studied topic in recent years because it is of great importance to both chiral science and supramolecular chemistry, where pillar[*n*]arenes are emerging as a promising class of planar chiral macrocyclic hosts for CPL. We herein synthesized an unusual planar chiral charge-transfer dye (P5BB) by covalent coupling of triarylborane (Ar_3_B) as an electron acceptor to parent pillar[5]arene as an electron donor. The intramolecular charge transfer (ICT) nature of P5BB not only caused a thermally responsive emission but also boosted the luminescence dissymmetry factor (*g*_lum_). Interestingly, the specific binding of fluoride ions changed the photophysical properties of P5BB, including absorption, fluorescence, circular dichroism (CD), and CPL, which could be exploited as an optical probe for multi-channel detection of fluoride ions. Furthermore, the chiroptical changes were observed upon addition of 1,4-dibromobutane as an achiral guest.

## Abstract 2

Pillar[5]arene-based charge transfer dyes show bright circularly polarized luminescence. The intramolecular charge transfer from pillar[5]arene to triarylborane induces an approximately 10-fold increase in luminescence dissymmetry factors.

## Graphical abstract

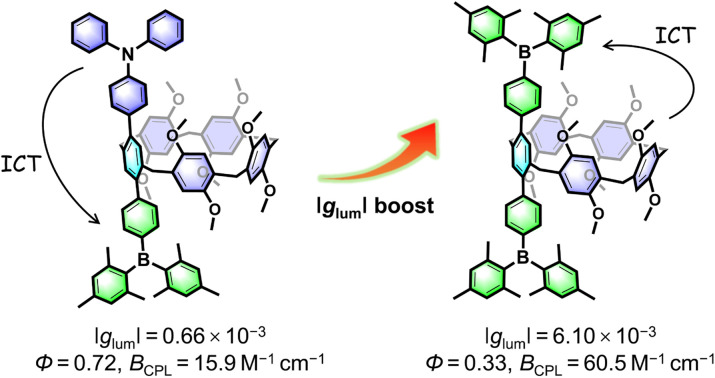

## Introduction

Chirality, as one of the most significant phenomena, is ubiquitous in life and the environment, and determines the daily physiological activities and metabolism of life.^1^ Chiral science unarguably promotes the development of life, medicine and materials science.^2^ Chiroptical functional materials with circularly polarized luminescence (CPL) have drawn great attention in the last decade,^3^ not only for the understanding of the inherent principles of chirality but also owing to their wide potential applications in chiral sensing,^4^ photoelectric devices,^5^ 3D displays,^6^ asymmetric catalysis^7^ and so forth. Generally, CPL is generated due to the molecules or supramolecular aggregates having both chiral features and luminescent properties.^8^ Therefore, it has become an effective strategy to achieve CPL activity by connecting luminophores with chiral fragments (*e.g.*, binaphthyls, helicenes, and [2.2]paracyclophane).^9^ Recently, CPL-active systems based on planar chiral analogues have attracted increasing attention,^10^ because their inherent macrocyclic skeletons have more important potential in the field of chiral supramolecular chemistry (*e.g.*, chiral network gelation and chiral host–guest recognition).

**Scheme 1:**
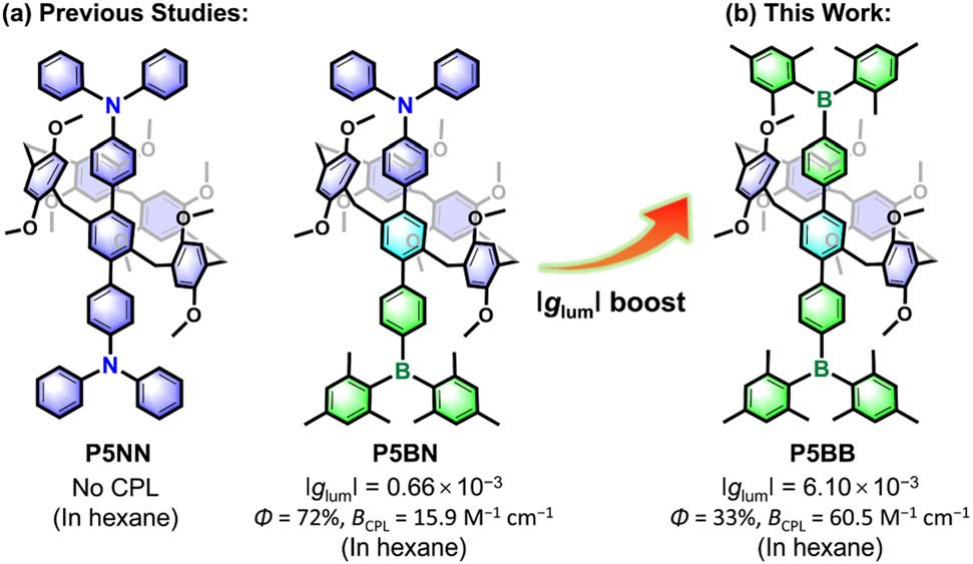
Research foundation and design strategy of pillararene-based planar chiral charge transfer dye with amplified *g*_lum_ values.

Pillararenes,^11^ as an important type of macrocyclic arene, have attracted extensive studies and made significant contributions in host–guest recognition and self-assembly because of their unique structure and easy synthesis.^12^ Pillararenes possess planar chirality, which comes from the different orientations of 1,4-alkoxyphenyl units.^13^ Although the enantiomers (p*S* and p*R*) are easy to interconvert due to the dynamic rotations of phenyl units, stable chiral configurations can still be achieved by reasonable molecular functionalization of the parent pillararenes. For example, introducing 10 cyclohexylmethyl groups at both rims of pillar[5]arene could prevent the rotations of benzene rings.^14^ Stoddart *et al.* also developed an effective strategy to obtain separable enantiomers by introducing bulky π-conjugated units at the A1/A2 positions of pillar[5]arene.^15^ Recently, pillararenes have been used to prepare CPL-active molecules by integrating with appropriate fluorophores.^16^ For example, Chen *et al.* reported two π-conjugated CPL-active systems (P5NN and P5BN) through axial functionalization of pillar[5]arene with sterically bulky triarylamine (Ar_3_N) and triarylborane (Ar_3_B) (Scheme 1); however, the *g*_lum_ values were only a 10^−4^ order because the luminescence largely depended on axial π-conjugated fluorophores and hence limited the transfer of chirality.^17^ In 2022, Ogoshi *et al.* reported a series of rim-differentiated C_5_-symmetric pillar[5]arenes with improved *g*_lum_ values, but it was difficult to obtain proper fluorescence efficiency while improving the asymmetry factor.^18^ Therefore, the design and synthesis of CPL-active pillararenes at the molecular level with a good balance between *g*_lum_ factors and luminescence efficiency are highly anticipated.

Theoretically, the *g*_lum_ factor is simply approximated using 4|*m*|cos *θ*/|*μ*|, where *m*, *μ* and *θ* are the magnetic transition dipole moment, electric transition dipole moment and the angle between *m* and *μ*, respectively.^19^ If organic systems are appropriately designed to have weaker electric dipole transition and stronger magnetic dipole transition, it could lead to CPL-active materials with high *g*_lum_. In fact, a large number of chiral organic molecules show relatively low *g*_lum_ values because of their electric dipole-allowed but magnetic dipole-forbidden transitions.^20^ In contrast, because charge transfer (CT) systems possess relatively small |*μ*| and large |*m*|, the larger *g*_lum_ values obtained from the CT state are hypothesized.^21^ As a typical electron acceptor, Ar_3_B has extensive application prospects in organic optoelectronic materials and stimulus-responsive materials.^22^ Notably, Ar_3_B are enabled to show distinctive CT emission once they are electronically coupled with electron donors.^23^ Pillararenes possess electron rich macrocyclic structures and can be used as a kind of electron donor. As one of our continuous pursuits of functionalized pillar[5]arenes,^24^ we herein propose a facile methodology to amplify *g*_lum_ values through functionalizing the pillar[5]arene parent to construct planar chiral CT dye (P5BB) with Ar_3_B. Sterically bulky Ar_3_B fluorophores not only allow the enantiomeric resolution but also promote intramolecular charge transfer (ICT) from pillar[5]arene to Ar_3_B. Based on the inherent host–guest nature of pillar[5]arene and the stimulus-responses of Ar_3_B, the chiroptical response behaviors of this chiral system were further studied. The details are presented herein.

## Results and discussion

**Scheme 2:**
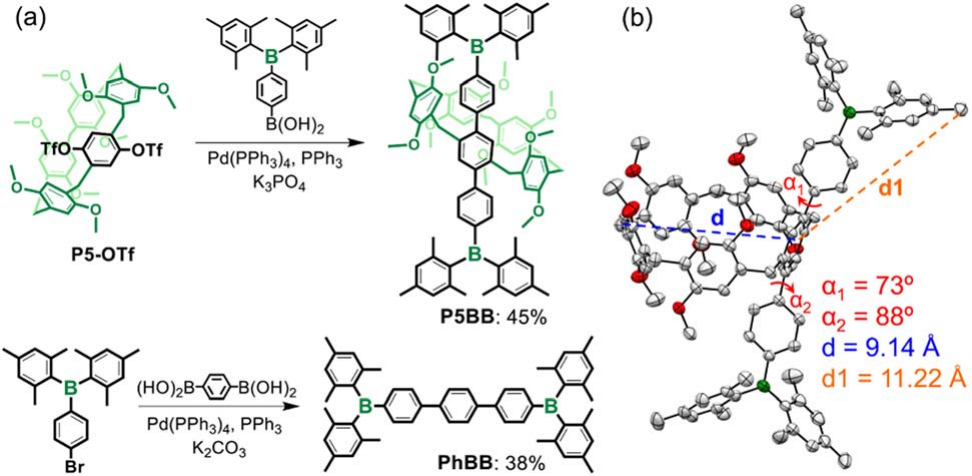
(a) Synthetic approach of P5BB and PhBB. (b) X-ray crystal structure of P5BB in elemental colors (C: grey, O: red, B: green; ellipsoid probability = 50%). All the hydrogen atoms and solvent molecules are omitted for clarity.

The key synthetic process of P5BB is shown in Scheme 2a and the ESI.† The core planar chiral block P5-OTf was directly obtained *via* the previous report,^25^ and then Pd-catalyzed Suzuki coupling by the reaction of P5-OTf with 2.0 equiv. Mes_2_B-containing phenylboronic acid led to the formation of P5BB in 45% yield. Similarly, PhBB was also obtained by standard Suzuki coupling of (4-bromophenyl)dimesitylborane with 1,4-phenylenediboronic acid in 38% yield. The chemical structures of P5BB and PhBB were fully characterized by ^1^H, ^13^C, and ^11^B NMR and high-resolution mass spectrometry (HRMS). A single crystal of *rac*-P5BB for X-ray diffraction analysis was collected by slowly evaporating the solution of acetone/MeOH (v/v = 1 : 1). In the crystalline form, a highly twisted π-conjugated skeleton was observed, as confirmed by dihedral angles (*α*_1_ and *α*_2_) that are measured to be 73° and 88°, respectively (Scheme 2b). Molecular size measurement revealed that the Ar_3_B-substituents (*d*_1_ = 11.22 Å) are greater than the cavity diameter of pillar[5]arene (*d* = 9.14 Å), so the racemization of enantiomers is sufficient to be inhibited through Ar_3_B-substituents. The equimolar enantiomers of p*S*-P5BB and p*R*-P5BB are packed in a unit cell *via* C–H⋯O, C–H⋯π and C–H⋯C interactions (Fig. S8†).

**Fig. 1:**
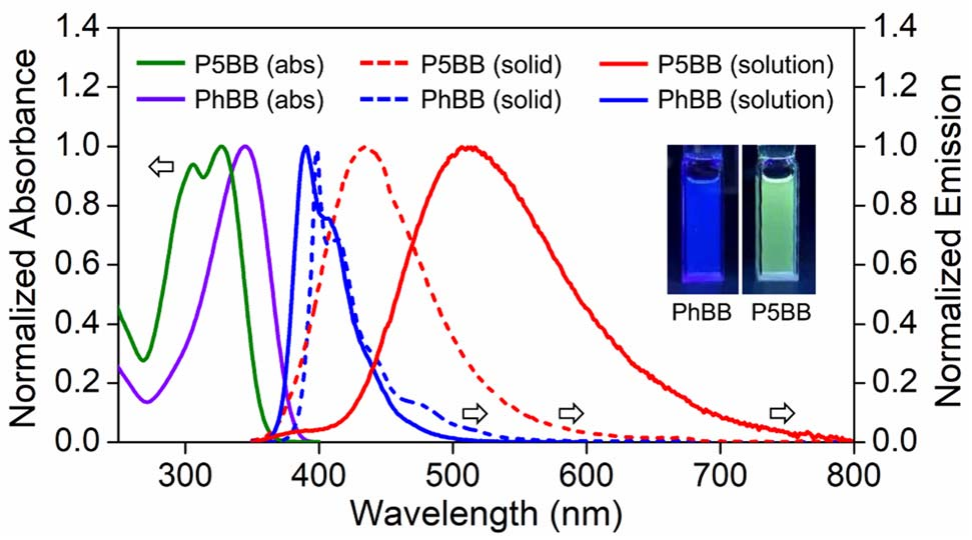
UV-vis absorption and emission spectra of *rac*-P5BB (*λ*_ex_ = 306 nm) and PhBB (*λ*_ex_ = 345 nm): solid line, in THF (*c* = 1.0 × 10^−5^ mol L^−1^); dashed line and solid state. Inset: photographs showing the emission colors of *rac*-P5BB and PhBB in THF under 365 nm UV irradiation.

The photophysical properties of P5BB and PhBB were investigated in THF solution and the solid state (Fig. 1 and Table 1). PhBB shows a strong absorption band of π–π* transition at 345 nm. However, P5BB exhibits two absorption peaks at 306 nm and 328 nm, corresponding to the local π–π* transitions in the pillar[5]arene motif and in the axial conjugated skeleton, respectively (Fig. S9†).^22*i*^ In comparison to PhBB, P5BB showed significantly red-shifted emission in both the solid state and solution, which was ascribed to the ICT between the pillar[5]arene donor and Ar_3_B acceptor. The ICT nature could be verified by a visible solvatochromic emission in various polar solvents (Fig. S11†). Owing to the temperature dependence of the equilibrium between the local excited (LE) state and the ICT excited state,^26^ we explored the thermally responsive emission of P5BB in 2-methyltetrahydrofuran as a low melting point solvent (Fig. S12†). At low temperature (150 K), P5BB showed a significantly dual emission band with a main ICT emission at 552 nm (*τ* = 33.9 ns) slightly overlapped with a minor LE emission at 405 nm (*τ* = 2.3 ns). With temperature increasing from 150 to 330 K, the main emission band of P5BB experienced an apparent hypsochromic shift, and the emission color change from yellow to blue was monitored using CIE coordinates. There is a good linear relationship of the maximum emission wavelength *versus* temperature with a correlation coefficient of 0.976. It is noteworthy that the above thermochromic response is completely reversible. The good accuracy and reversibility suggested that the system is an ideal candidate for high-performance fluorescent thermometers.

**Table 1:** Photophysical, electrochemical and computational data of P5NN, P5BN, P5BB and PhBB

	*λ*_abs_ a (nm)	*λ*_em_ a (nm)	*Φ*_L_ b (%)	*Φ*_S_ c (%)	*τ*_ave_ d (ns)	*E*_HOMO_ e (eV)	*E*_LUMO_ e (eV)	*E*_gap(DFT)_ f (eV)	*E*_ox_ g (V)	*E*_red_ g (V)
P5NN^h^	306	393	61	19	1.2	−4.88	−0.64	4.24	+0.52	—
P5BN^h^	303, 330	493	99	57	6.4	−4.89	−1.67	3.22	+0.59	−2.49
P5BB	306, 328	510	12	16	20.5	−4.91	−1.77	3.14	+0.61	−2.46
PhBB	345	390	99	70	1.8	−5.83	−1.94	3.89	—	—

a Measured in THF (1.0 × 10^−5^ mol L^−1^) at room temperature.

b Fluorescence quantum yield (*Φ*_L_) measured in THF.

c *Φ*_S_ measured in the solid state.

d Average fluorescence lifetime in THF at room temperature.

e HOMO and LUMO energy levels obtained using DFT calculations (B3LYP, 6-31G(d,p)).

f *E*_gap_(DFT) = *E*_LUMO_ − *E*_HOMO_ (B3LYP, 6-31G(d,p)).

g The first half-wave potentials of oxidation and reduction processes.

h See ref. 17.

In order to further understand the correlation between the molecular structures and the photophysical properties of P5BB and PhBB, the electronic structure calculations were performed using DFT (B3LYP, 6-31G(d,p)) and TD-DFT (B3LYP, 6-31G(d)). TD-DFT computations revealed that the absorption of PhBB is mainly attributed to the π–π* transition to the S_1_ state (HOMO → LUMO, *f* = 1.2994) (Fig. S14 and Table S5†). In P5BB, the HOMO is fully located on the electron-donor pillar[5]arene backbone; however, the LUMO is delocalized over the B-conjugated π-extension (Fig. S15†). By means of TD-DFT calculations, it was found that the first three CT transitions are the results of vertical excitations from the pillar[5]arene-localized HOMO, HOMO−1 and HOMO−2, to the LUMO (Fig. S16†). The higher excited states (S_4_ and S_5_) are excitations to the LUMO+1 level from the HOMO and HOMO−1. Cyclic voltammetry (CV) of P5BB showed a reversible reduction potential at −2.46 V (*vs.* Fc+/Fc, in THF), representing the reduction of the electron-deficient Ar_3_B segments (Fig. S17†). Three reversible oxidation curves with the first oxidation potentials at +0.61 V for P5BB (*vs.* Fc+/Fc, in CH_2_Cl_2_) were identified, corresponding to the oxidation of the pillar[5]arene skeleton. The electrochemical gap (3.07 eV) is almost consistent with the HOMO–LUMO gap (3.14 eV) *via* the DFT calculations. As expected, the first oxidation potential of P5BB is slightly higher than that of compounds with the N donor (P5NN, +0.52 V; P5BN, +0.59 V), which is completely consistent with the slightly lower HOMO energy level (P5BB, −4.91 eV; P5NN, −4.88 eV; P5BN, −4.89 V).

**Fig. 2:**
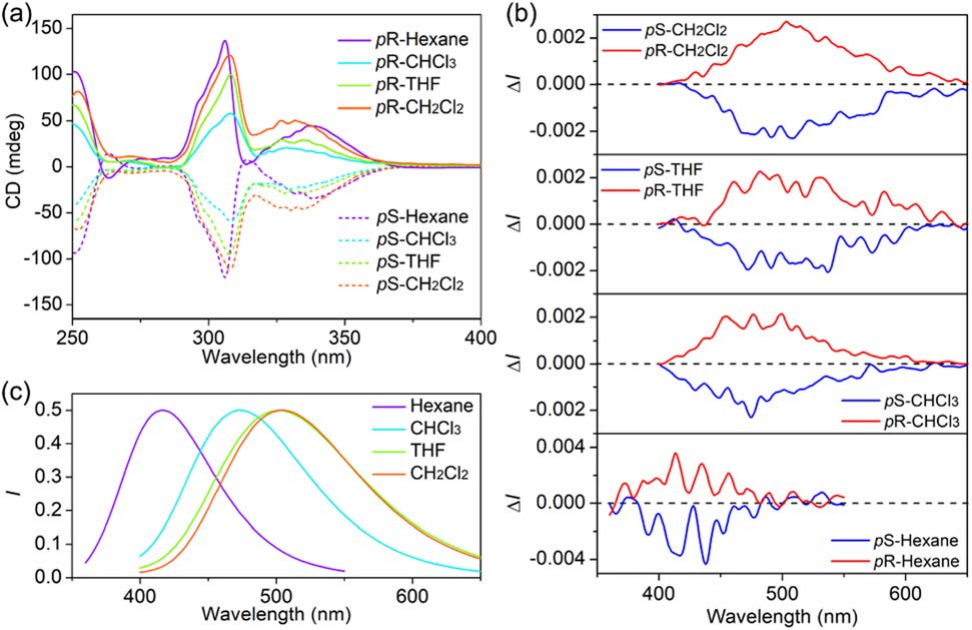
(a) CD, (b) CPL and (c) fluorescence spectra of enantiomers of P5BB in different solvents (*c* = 5.0 × 10^−5^ mol L^−1^, *λ*_ex_ = 306 nm).

The definite enantiomeric configuration inspired us to prepare their optically pure forms for studying the chiroptical properties. Two separated peaks with a 1 : 1 area were observed by the initial injection of *rac*-P5BB into a chiral HPLC with a Daicel Chiralpak IB N-5 column (hexane/2-propanol = 96/4, v/v). After each fraction was well isolated with an enantiomeric excess (>99% ee, Fig. S18†), the CD spectra of the enantiomers exhibited mirror-image relationships with strong CD absorption peaks in various organic solvents (Fig. 2a). The CD signals belonging to pillar[5]arene cores are clearly observed at 310 nm,^27^ and the absorption dissymmetry factor |*g*_abs_| was calculated to be 1.53 × 10^−3^ in hexane. On the basis of a comparison of the experimental CD absorption signals with the results of previous reports,^27^ the p*S* configurations correspond to the first peak and the second peak correspond to p*R* configurations in HPLC traces. In the CPL spectra, the enantiomers exhibited almost mirror-imaged signals in various solvents (Fig. 2b). The |*g*_lum_| reached 10^−3^ in solution as well as in the solid state (Table 2), which is significantly higher than that of P5NN and P5BN. The CPL spectra of P5BB gradually redshifted with the increase in solvent polarity, and the positions of CPL signals were almost consistent with that of fluorescence. In fact, the enantiomers of P5NN and P5BN did not show apparent CPL signals in various solvents (except for hexane), suggesting that the chiral transfer did not occur effectively in this case. These phenomena indicated that the ICT character of P5BB not only amplifies the *g*_lum_ factors but also adjusts the color of CPL *via* the selection of different solvents. With all the necessary photophysical and chiral optical data in hand, the CPL brightness (*B*_CPL_: defined as *B*_CPL_ = *ε* × *Φ* × |*g*_lum_|/2) in solutions was further calculated to evaluate the overall performance of the CPL dyes. The *B*_CPL_ of p*S*-P5BB was calculated to be 60.5 M^−1^ cm^−1^ in hexane, which is significantly higher than that of p*S*-P5BN (15.9 M^−1^ cm^−1^), indicating that P5BB possesses excellent chiroptical properties for future CPL applications.

**Table 2:** The chiroptical property data of P5NN, P5BN and P5BB

		*λ*_abs_ (nm)	*ε* a (M^−1^ cm^−1^)	*λ*_em_ (nm)	*Φ* b (%)	*g*_lum_ c (10^−3^)	*B*_CPL_ d (M^−1^ cm^−1^)
p*S*-P5NN^e^	Hexane	306	7.31 × 10^4^ (306)	391	52	—	—
	Solid	—	—	398	19	+0.22	—
p*S*-P5BN^e^	Hexane	303, 330	6.71 × 10^4^ (303)	403	72	+0.66	15.9
	Solid	—	—	455	57	+0.88	—
p*S*-P5BB	Hexane	305, 329	6.01 × 10^4^ (305)	422	33	−6.10	60.5
	CHCl_3_	308, 328	5.98 × 10^4^ (308)	473	23	−3.64	25.1
	THF	306, 328	6.08 × 10^4^ (306)	510	12	−3.34	12.2
	CH_2_Cl_2_	306, 327	6.41 × 10^4^ (306)	512	12	−4.62	17.8
	Solid	—	—	435	16	−5.13	—
p*S*-P5BB + G	CHCl_3_	308, 328	6.00 × 10^4^ (308)	460	18	−4.16	22.4
p*S*-P5BB + F^−^	THF	295	3.35 × 10^4^ (295)	365	56	−5.08	47.6

a Molar absorption coefficient (*ε*) at a given wavelength.

b Fluorescence quantum yield (*Φ*).

c Luminescence dissymmetry factor (*g*_lum_).

d The CPL brightness (*B*_CPL_).

e See ref. 17.

**Fig. 3:**
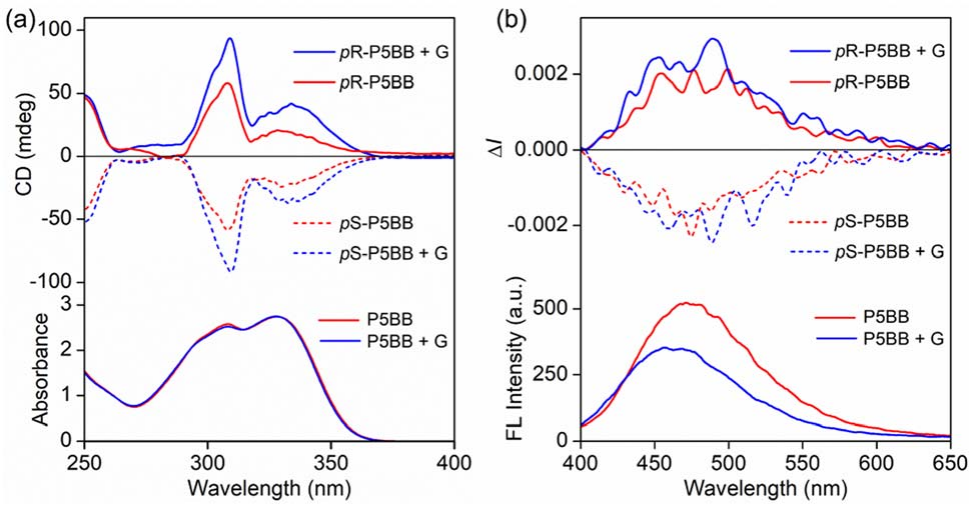
(a) CD and UV-vis absorption spectra and (b) CPL and fluorescence spectral responses of enantiomers for P5BB in CHCl_3_ (*c* = 5.0 × 10^−5^ mol L^−1^, *λ*_ex_ = 306 nm) upon addition of 50 equivalents of 1,4-dibromobutane G as a neutral guest.

In order to further evaluate the chiroptical changes induced through host–guest chemistry, we selected the neutral small molecule 1,4-dibromobutane (G) as the representative guest to bind P5BB by 1 : 1 complexation.^28^ As shown in Fig. S19,† upon the addition of excess 1,4-dibromobutane to P5BB in CDCl_3_ solution, the proton signals of H_1–3_ and H_5–11_ on P5BB were shifted downfield, and meanwhile, all signal peaks on the guest were found to shift upfield in ^1^H NMR spectra. Additionally, the proton peaks of the guest were substantially reduced after complexation due to inclusion-induced shielding effects. The association constant (*K*_a_) was determined to be 187.8 M^−1^ using the nonlinear data fitting of ^1^H NMR titrations (Fig. S20 and S21†). This implies that host–guest recognition between P5BB and 1,4-dibromobutane has occurred. Notably, the addition of a guest results in dramatic enhancement of CD signals at 310 nm and 328 nm in CHCl_3_, while the absorption does not change significantly, implying that the host–guest complexation decreases the configuration rotation of the pillar[5]arene skeleton of P5BB (Fig. 3). The addition of excessive 1,4-dibromobutane led to a decrease in fluorescence intensity, and meanwhile, the CPL signals showed a mild enhancement, indicating the host–guest recognition further affected the excited state chiral conformation of P5BB.

**Fig. 4:**
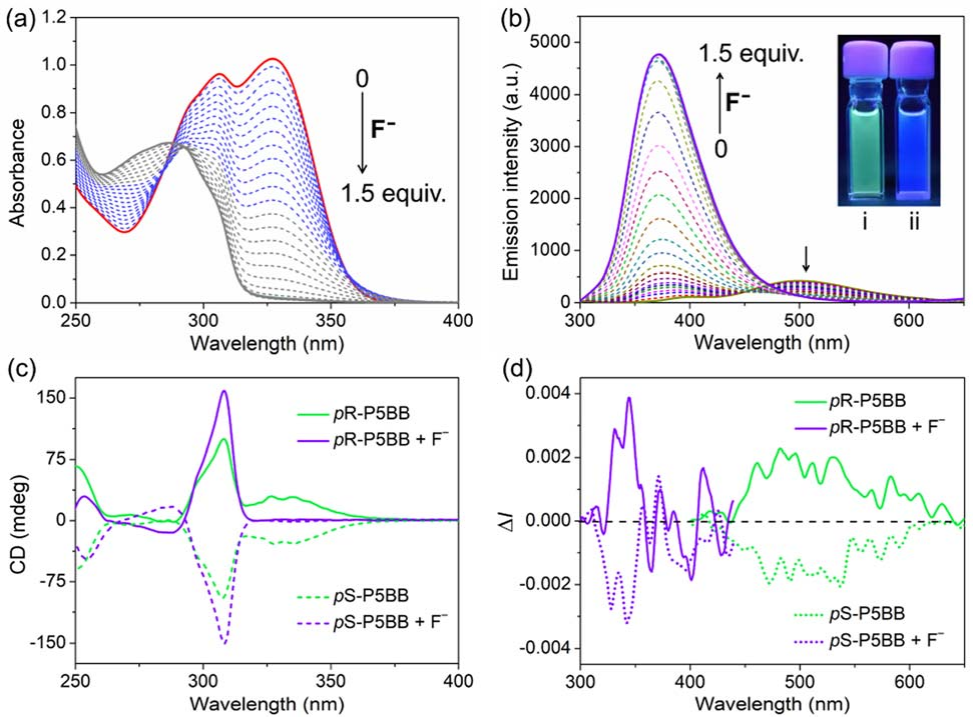
(a) UV-vis absorption and (b) emission spectra with the addition of different concentrations of F^−^ anions to *rac*-P5BB in THF solution (*c* = 2.0 × 10^−5^ mol L^−1^, *λ*_ex_ = 306 nm). Inset: photographs of solutions before (i) and after (ii) the addition of F^−^ (1.5 equiv.) under 365 nm UV irradiation. (c) CD and (d) CPL spectral responses of enantiomers for P5BB (*c* = 5.0 × 10^−5^ mol L^−1^, *λ*_ex_ = 306 nm) in the presence of 3.0 equiv. of F^−^ anions.

Considering the Lewis acidity of Ar_3_B, another interesting thing is the response behavior of P5BB toward small Lewis bases.^29^ Herein, the optical responses of four common halogen anions (including F^−^, Cl^−^, Br^−^ and I^−^) as Lewis bases to P5BB were primarily investigated in THF solution. In the UV-vis absorption spectra (Fig. 4a), the absorption bands at around 306 nm and 328 nm of P5BB gradually decrease with the addition of F^−^ anions, which is due to the fact that the conjugation of the Ar_3_B moiety was broken by the formation of tetra-coordinated boron complexing with F^−^ anions (Scheme S1 and Fig. S24†). The limit of detection (LOD) was further calculated to be 43.3 nM based on the 3*σ*/*S* values (Fig. S22†). Remarkably, other halogen ions (Cl^−^, Br^−^ and I^−^) could not cause any significant changes in absorption spectra, which was likely ascribed to the smaller steric size of the F^−^ anion. Furthermore, the strong affinity between fluoride and boron also played a crucial role. As displayed in Fig. 4b, as the concentration of F^−^ increased, the emission peak at 510 nm of P5BB slowly declined, while the emission peak at 365 nm rapidly enhanced. Quite evidently, the emission spectrum exhibited a blue shift (∼145 nm) with the emission color changing from green to purple, and the fluorescence intensity increased dramatically with *Φ* up to 56%. These phenomena indicated that the binding of F^−^ anions with the Ar_3_B unit prevented the ICT process in the excited state, leading to the enhancement of LE-state emission.^29^ Moreover, in the presence of an excess amount of F^−^ anions, the CD signals of the enantiomers at 328 nm disappeared, while the signals at 310 nm were effectively enhanced. Even after complexation with F^−^ anions, significant CPL signals with a blue-shift were also detected. The |*g*_lum_| values were calculated to reach 5.08 × 10^−3^ with a *B*_CPL_ of 47.6 in this case and were much higher than those of chiral pillararene derivatives reported in ref. 16b (Scheme S2†). These investigations confirmed that the P5BB system could realize UV-vis absorption/fluorescence/CD/CPL quadruple-mode sensing of F^−^ anions.

## Conclusions

In summary, we have developed an effective strategy to design and synthesize a π-conjugated planar chiral CT dye P5BB through integration of pillar[5]arene with organoborane. The ICT character in P5BB not only amplified the *g*_lum_ values but also could tune the CPL color by the selection of various solvents with different polarities. Approximately 10-fold enhancements in *g*_lum_ were observed from P5BB to P5BN in hexane, which was ascribed to the fact that the photo-responsive unit itself contains planar chiral pillar[5]arene in P5BB. Inclusion of the achiral guest 1,4-dibromobutane directly enhanced chiral optical signals, including CD and CPL. Furthermore, the coordination of F^−^ anions with boron leads to remarkable changes in absorption, fluorescence, CD and CPL signals of the P5BB system. Consequently, the P5BB platform could specifically detect F^−^ anions with high sensitivity and favorable selectivity. The current design strategy to fabricate a CPL-active ICT dye with the amplification of the *g*_lum_ factor is expected to promote the development of future planar chiral pillararene materials. We envision that this work will catalyze the future application of pillararene-based CPL-active systems in chiroptical sensing, chiral supramolecular chemistry and CPL-based photoelectric devices.

## Data availability

All the data supporting this article have been included in the main text and the ESI.†

## Author contributions

J.-F. C. initiated and coordinated the study. J.-F. C., Q.-X. G. and T.-B W. designed all experiments, analyzed the data, and wrote the manuscript. L. L. carried out the computational work. P. C. and T.-B. W. supervised and administrated the project. All authors approved the final version.

## Conflicts of interest

There are no conflicts to declare.

## Supplementary Material

SC-014-D2SC06000K-s001

SC-014-D2SC06000K-s002
