# Supplementary material for: A pillar[5]arene-based planar chiral charge-transfer dye with enhanced circularly polarized luminescence and multiple responsive chiroptical changes
Source: Chem Sci. 2023 Jan 3;14(4):987–93. doi: 10.1039/d2sc06000k (PMC9890741; doi:10.1039/d2sc06000k)
Supplement: SC-014-D2SC06000K-s001 [file SC-014-D2SC06000K-s001.pdf]

Electronic Supplementary Material (ESI) for Chemical Science.
This journal is © The Royal Society of Chemistry 2023

Pillar[5]arene-based planar chiral charge-transfer dye with enhanced circularly polarized luminescence and multiple responsive chiroptical changes

Jin-Fa Chen,* Qing-Xiu Gao, Lijie Liu, Pangkuan Chen and Tai-Bao Wei

Key Laboratory of Eco-Environment-Related Polymer Materials, Ministry of Education of China; Key Laboratory of Polymer Materials of Gansu Province; College of Chemistry and Chemical Engineering, Northwest Normal University, Lanzhou, Gansu 730070, P. R. China.

*chenjinfa@nwnu.edu.cn

Table of Contents

1. Experimental Section S2
2. Synthetic Procedures S4
3. Characterization by NMR and HRMS Spectroscopy S6
4. X-ray Structure Determination S10
5. Photophysical Measurements S11
6. DFT and TD-DFT Computations S13
7. Electrochemical Measurements S15
8. Chiral HPLC Trace Analysis S16
9. Host–Guest Recognition S17
10. Anion Recognition S19
11. Coordinates (Å) for the Optimized Structures S21
12. References S24

1. Experimental Section

Materials and Methods: 1,4-Phenylenediboronic acid, $Pd(PPh_3)_4$, PPh_3, K_2CO_3 and K_3PO_4 were purchased from Energy Chemical. Dichloromethane, petroleum ether, hexane, ethanol, toluene, 1,4-dioxane, acetone and ethyl acetate were purchased from Sinopharm Chemical Reagent Co., Ltd.

400 MHz ^1H, 101 MHz ^{13}C, and 225 MHz ^{11}B NMR spectra were recorded on a Bruker spectrometer. ^{11}B NMR spectra were acquired with boron-free quartz NMR tubes and the spectra were referenced externally to $BF_3{\cdot}Et_2O$ (δ = 0). High resolution mass spectral data were obtained *via* ESI on an Agilent (Q-TOF 6520) analyzer.

Dissolve 5 mg **P5BB** in acetone (0.5mL)/methanol (0.5 mL) solvent, then slowly evaporate at room temperature to get colorless bulk crystals. Single crystal X-ray data were obtained on a Bruker D8 X-ray single crystal Venture diffractometer using Mo Kα radiation (λ = 0.71073 Å). SAINT5.0 and SADABS programs are used for the reduction and absorption correction of crystal data. The resolution and refinement of the crystal structure are obtained on the SHELXTL-97 software. Using the direct or Patterson methods, all non-hydrogen source coordinates are obtained by using the differential Fourier method and the least square method. Then the geometric method and the difference value are used. The hydrogen atom coordinates were obtained by Fourier method, and the crystal structure was obtained. The CCDC number of 2215838 (**P5BB**) is deposited.

UV-visible absorption spectra were recorded on a Cary 300 UV-Vis spectrophotometer. The fluorescence spectra were recorded on a Lengguang Tech F97 Pro spectrophotometer. The fluorescence lifetime data was measured on an Edinburgh Instruments FLS980 spectrophotometer. Fluorescent quantum efficiencies were determined using a Hamamatsu Quantaurus-QY spectrometer (C11347-11). The CD spectra were measured using a Circular Dichroism spectrometer (J-1500, Jasco). CPL measurements were performed with circularly polarized luminescence spectrometer (CPL-300, Jasco). In the spectral tests, the solid-state samples involved here are amorphous.

Electrochemical measurements were conducted on an AUTOLAB-CV-75W analyzer with a scan rate of 100 mV/s. The electrochemical cell was a standard three-compartment cell composed of a glass carbon working electrode, a Pt auxiliary electrode, and a Pt wire

reference electrode. All tests were performed using $[Bu_4N][PF_6]$ (0.1 M) as the supporting electrolyte. The voltammograms were obtained in anhydrous and nitrogen-saturated CH_2Cl_2 or THF solutions. The potentials are reported relative to the ferrocene/ferrocenium couple.

For titration experiments, fluorine ion solutions were prepared by dissolving the desired amount of solid $TBAF{\cdot}3H_2O$ in THF. Addition of fluorine ion to the sample solution was performed by a microsyringe (± 0.1 μL). Solutions of anions were prepared from the tetrabutylammonium salts of Cl^-, Br^-, and I^-.

DFT calculations were performed with the Gaussian 09 program. Geometry optimizations and vertical excitations were calculated by means of hybrid density functional B3LYP with the basis set of 6-31G(d,p). The input files and orbital representations were generated with Gaussview 5.0 (scaling radii of 75%, isovalue = 0.02). Excitation data were calculated using TD-DFT (B3LYP/6-31G(d)). The resulting structures were confirmed to be stationary points through vibrational frequency analysis.

2. Synthetic Procedures

2.1 Synthetic route of P5BB

Starting material **P5-OTf**[S1] and (4-(dimesitylboranyl) phenyl)boronic acid[S2] were prepared according to the literature procedures.

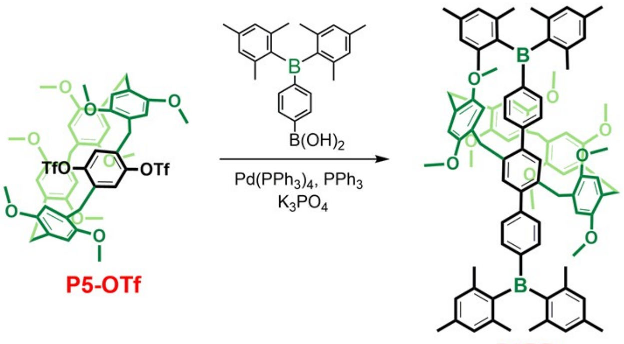

P5BB: The mixture of **P5-OTf** (196 mg, 0.2 mmol), (4-(dimesitylboranyl) phenyl)boronic acid (222 mg, 0.6 mmol), $Pd(PPh_3)_4$ (23 mg, 0.02 mmol), PPh_3 (13 mg, 0.05 mmol) and K_3PO_4 (106 mg, 0.5 mmol) in 1,4-dioxane/H_2O (4 mL/1 mL) was refluxed in an oil bath with stirring under N_2 for 72 h. The reaction mixture was then cooled to room temperature and poured into water (30 mL). After extraction with CH_2Cl_2, the combined organic phases were dried over Na_2SO_4. Purification by column chromatography on silica gel (petroleum ether/CH_2Cl_2/ethyl acetate = 100:1:2, v/v/v) gave **P5BB** as a white solid (120 mg, 45%). ^1H NMR (400 MHz, $CDCl_3$) δ 7.54 (d, J = 7.9 Hz, 4H), 7.29 (s, 2H), 7.20 (d, J = 7.9 Hz, 4H), 6.85 (s, 8H), 6.75 (s, 2H), 6.68 (s, 2H), 6.60 (s, 2H), 5.86 (s, 2H), 3.90–3.89 (m, 4H), 3.80 (s, 2H), 3.75–3.65 (m, 4H), 3.59 (s, 6H), 3.56 (s, 6H), 3.48 (s, 6H), 3.26 (s, 6H), 2.33 (s, 12H), 2.07 (s, 24H). ^{13}C NMR (101 MHz, $CDCl_3$) δ 151.1, 150.9, 150.7, 149.9, 146.4, 141.7, 140.8, 140.3, 138.7, 136.4, 136.3, 132.7, 129.4, 129.1, 128.6, 128.6, 128.2, 127.9, 122.7, 114.2, 114.1, 113.9, 55.9, 55.9, 55.8, 55.5, 32.3, 30.2, 23.5, 21.2, 20.4, 15.8. ^{11}B NMR (225 MHz, $CDCl_3$) δ 70 ppm. ESI-HRMS (m/z): calcd. for $C_{91}H_{100}B_2NO_8$ $[M+NH_4]^+$ 1356.7635, found 1356.7645.

2.2 Synthetic route of PhBB

Starting material (4-bromophenyl)dimesitylborane[S3] was prepared according to the literature procedures.

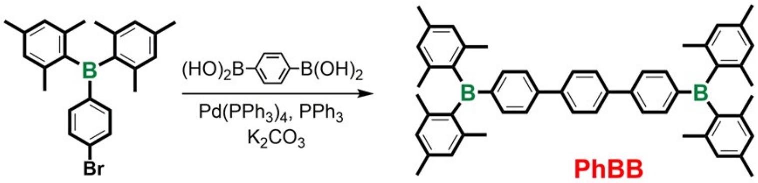

PhBB: The mixture of (4-bromophenyl)dimesitylborane (108 mg, 0.25 mmol), 1,4-phenylenediboronic acid (16.5 mg, 0.1 mmol), $Pd(PPh_3)_4$ (23 mg, 0.02 mmol), PPh_3 (13 mg, 0.05 mmol) and K_2CO_3 (138 mg, 1.0 mmol) in toluene/EtOH/H_2O (4 mL/0.5 mL/0.5 mL) was refluxed in an oil bath with stirring under N_2 for 48 h. The reaction mixture was then cooled to room temperature and poured into water (30 mL). After extraction with CH_2Cl_2, the combined organic phases were dried over Na_2SO_4. Purification by column chromatography on silica gel (petroleum ether/ethyl acetate = 200:1, v/v) gave **PhBB** as a white solid (28 mg, 38%). ^{1}H NMR (400 MHz, $CDCl_3$) δ 7.73 (s, 4H), 7.64–7.59 (m, 8H), 6.84 (s, 8H), 2.32 (s, 12H), 2.04 (s, 24H). ^{13}C NMR (101 MHz, $CDCl_3$) δ 144.7, 143.7, 141.7, 140.8, 140.0, 138.6, 137.1, 128.2, 127.6, 126.4, 23.5, 21.2. ^{11}B NMR (225 MHz, $CDCl_3$) δ 75 ppm.

3. Characterization by NMR and HRMS Spectroscopy

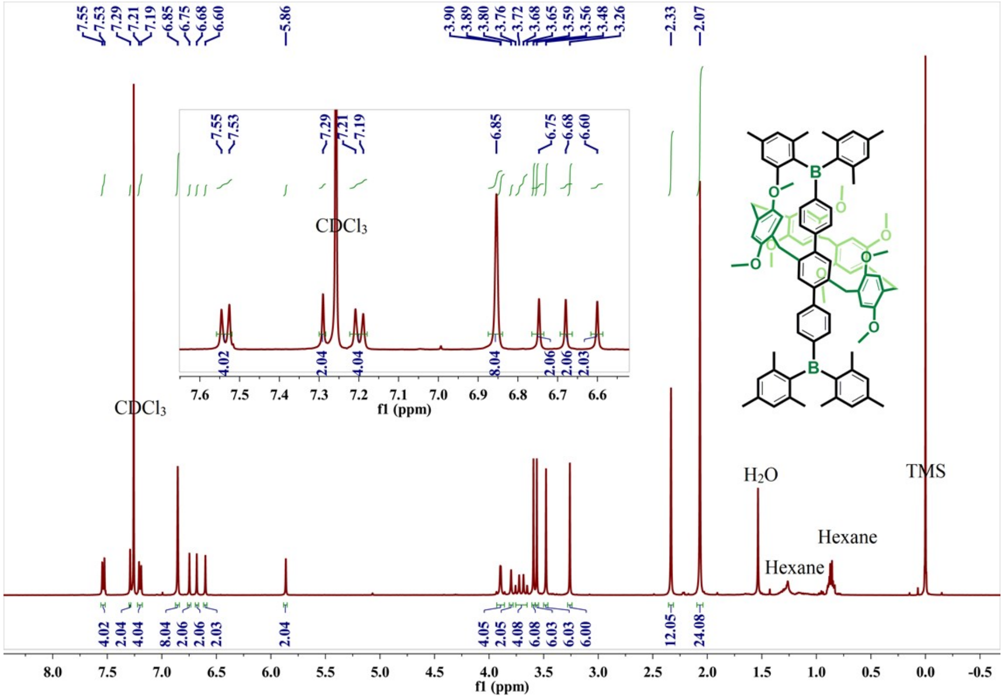

Figure S1. ^{1}H NMR (400 MHz, $CDCl_3$) spectrum of **P5BB**.

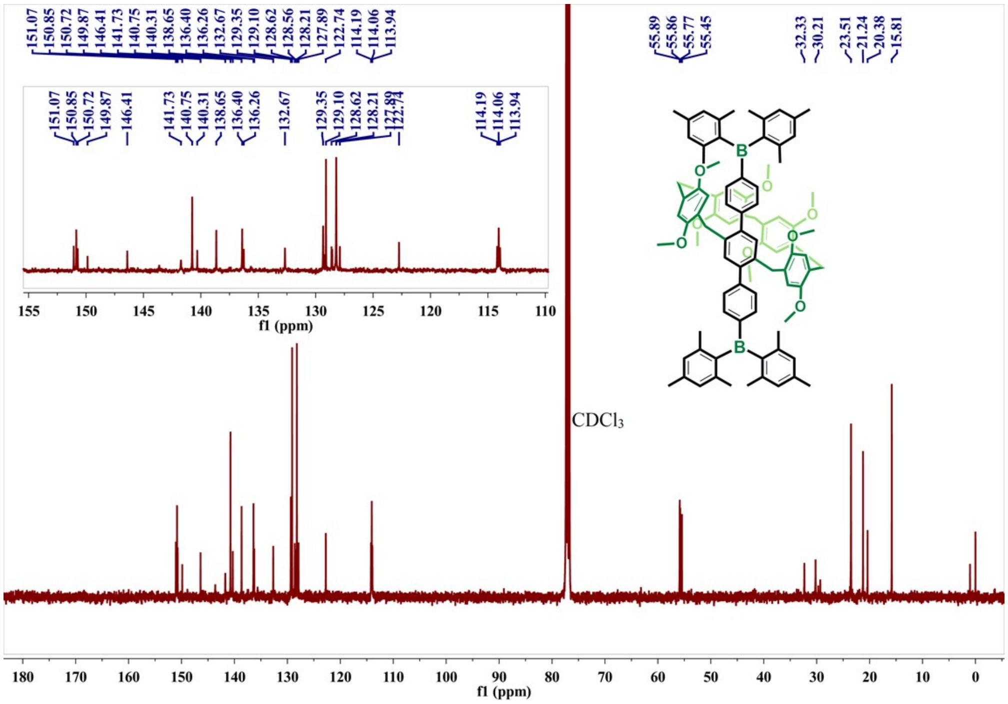

Figure S2. ^{13}C NMR (101 MHz, $CDCl_3$) spectrum of **P5BB**.

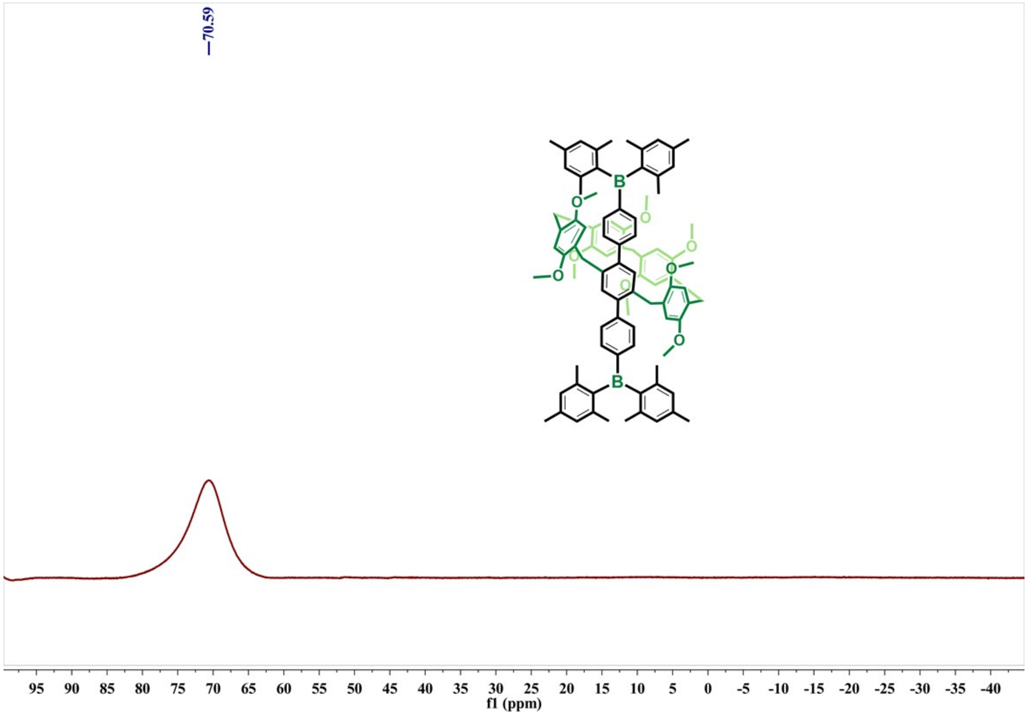

Figure S3. ^{11}B NMR (225 MHz, $CDCl_3$) spectrum of **P5BB**.

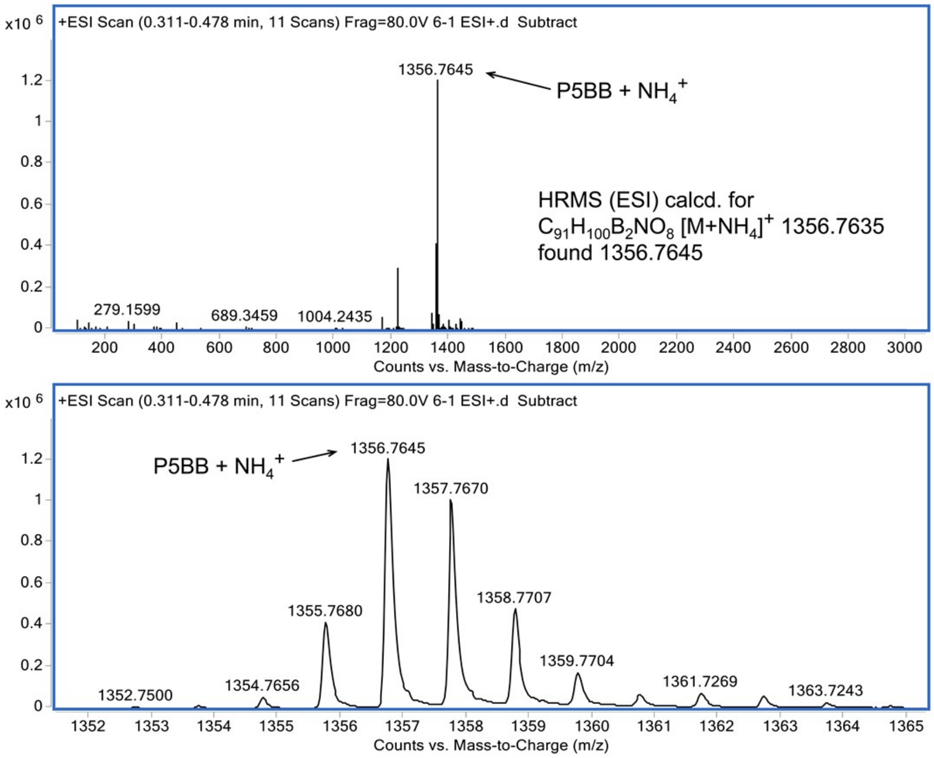

Figure S4. HRMS spectrum for **P5BB**.

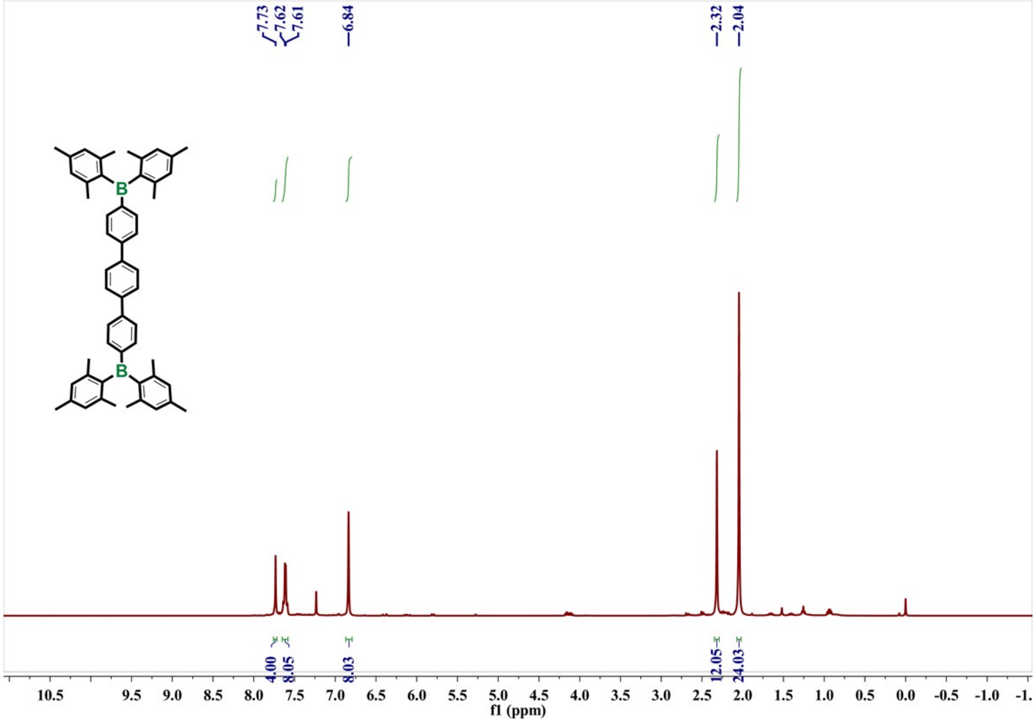

Figure S5. ^{1}H NMR (400 MHz, $CDCl_3$) spectrum of **PhBB**.

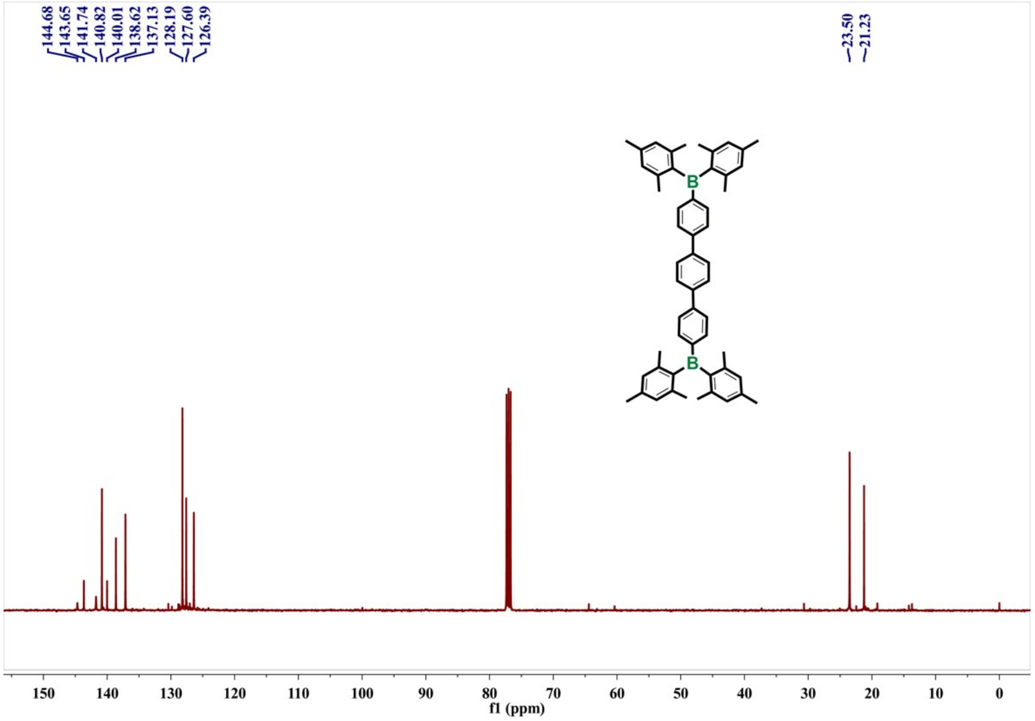

Figure S6. ^{13}C NMR (101 MHz, $CDCl_3$) spectrum of **PhBB**.

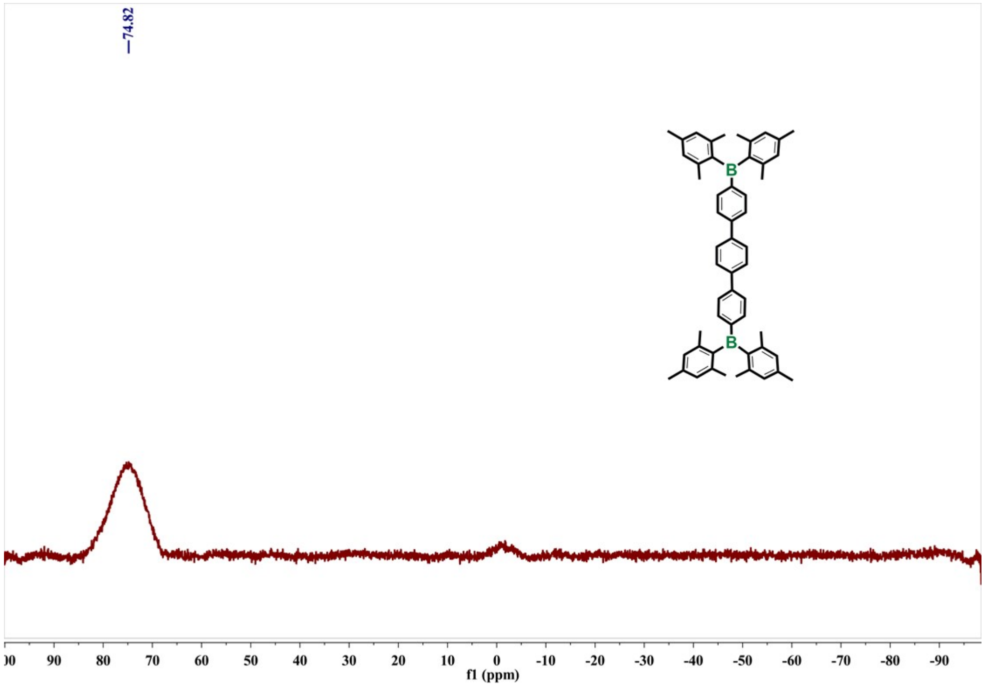

Figure S7. ^{11}B NMR (225 MHz, $CDCl_3$) spectrum of **PhBB**.

4. X-ray Structure Determination

Table S1. Crystallographic data and structure refinement for **P5BB**.

**Compound**	*Rac*-**P5BB**
Empirical formula	$C_{97}H_{106}B_2NO_{10}$
Formula weight	1453.43
Temperature/K	180.0
Crystal system	monoclinic
Space group	$P2_1/n$
Unit cell	a = 13.9414(11), b = 20.9320(17), c = 28.7875(18)<br>α = 90, β = 95.848(3), γ = 90
Volume/Å^3	8357.1(11)
Z	4
ρ_{calc}g/cm^3	1.155
μ/mm^{-1}	0.073
F(000)	3112.0
Crystal size/mm^3	0.3 × 0.3 × 0.09
Radiation	MoKα (λ = 0.71073)
2θ range for data collection/°	4.69–50
Index ranges	$-16 \leq h \leq 14$, $-24 \leq k \leq 24$, $-34 \leq l \leq 34$
Reflections collected	83864
Independent reflections	14710 [R_{int} =0.1085, R_{sigma} = 0.0727]
Data/restraints/parameters	14710/24/1038
Goodness-of-fit on F^2	0.990
Final R indexes [I>=2σ (I)]	R_1 = 0.0657, wR_2 = 0.1698
Final R indexes [all data]	R_1 = 0.1392, wR_2 = 0.1958
Largest diff. peak/hole (e. Å^{-3})	0.39/-0.29

Table S2. Selected bond distances (Å) from X-ray data for **P5BB**.

Bond	Bond Length	Bond	Bond Length
B(1)-C(1)	1.582(4)	B(2)-C(34)	1.560(4)
B(1)-C(10)	1.575(4)	B(2)-C(37)	1.563(4)
B(1)-C(19)	1.562(4)	B(2)-C(46)	1.574(4)

Table S3. Selected bond angles (°) from X-ray data for **P5BB**.

	Bond Angles		Bond Angles
C(1)-B(1)-C(10)	121.2(2)	C(34)-B(2)-C(37)	120.2(2)
C(1)-B(1)-C(19)	117.8(2)	C(34)-B(2)-C(46)	116.2(2)
C(10)-B(1)-C(19)	120.9(3)	C(37)-B(2)-C(46)	123.6(2)

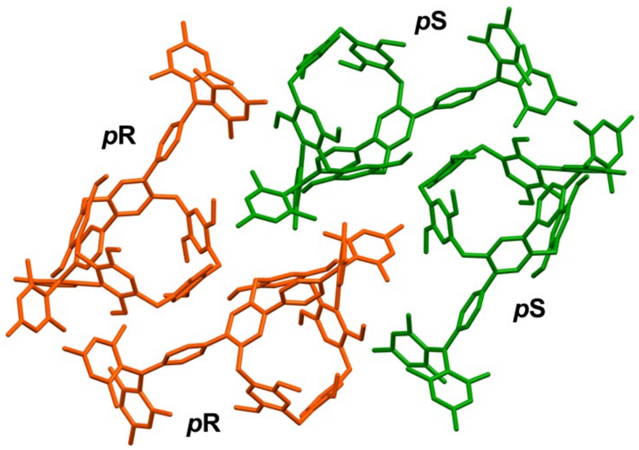

Figure S8. X-ray crystal stacks in *rac*-**P5BB** *via* C–H···π, C–H···O and C–H···C interactions. Enantiomers are highlighted in different colors. All the hydrogen atoms and solvent molecules are removed for clarity.

5. Photophysical Measurements

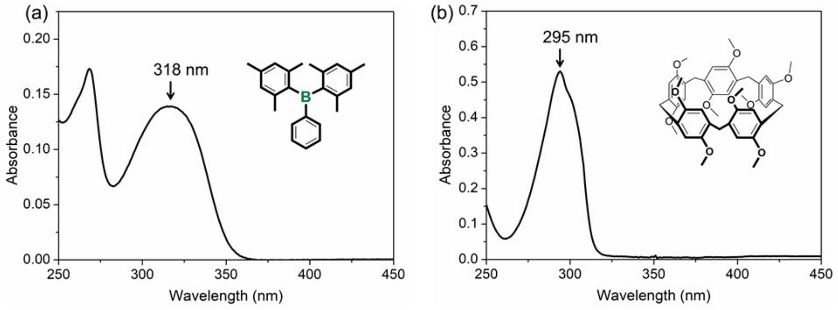

Figure S9. UV-vis absorption spectra of (a) triarylborane and (b) **DMP5** in THF (c = 1.0 × 10^{-5} M).

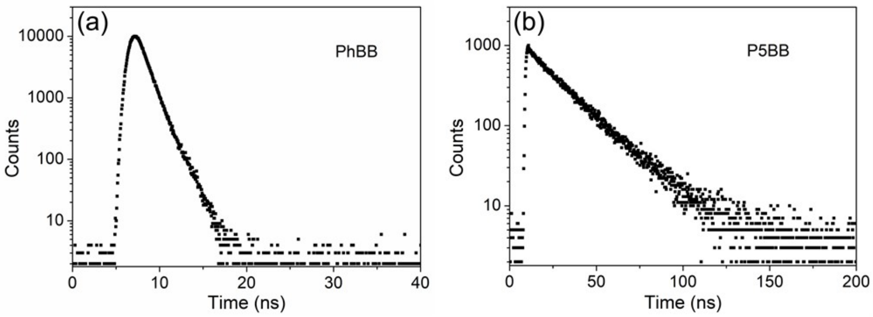

Figure S10. Transient photoluminescence decay spectra of (a) **PhBB** and (b) **P5BB** in THF (c = 1.0 × 10^{-5} M) at room temperature.

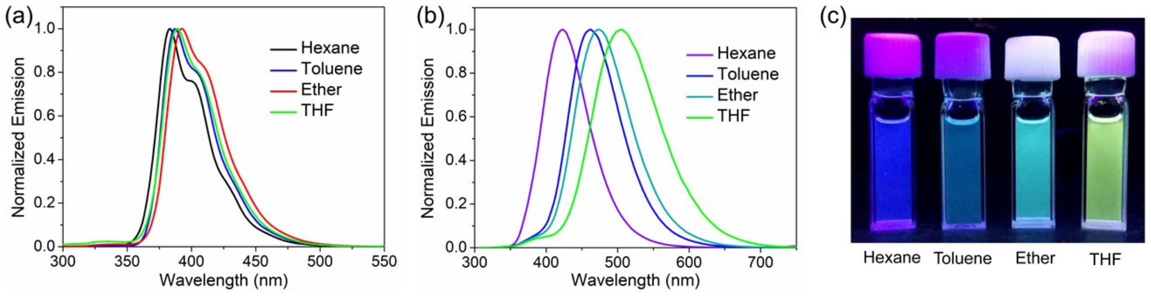

Figure S11. Emission spectra of (a) **PhBB** (λ_{ex} = 345 nm) and (b) **P5BB** (λ_{ex} = 306 nm) in different solvents ($c = 1.0 \times 10^{-5}$ M) with different polarity; (c) Photographs showing the emission colors of **P5BB** in different solvents under the 365 nm UV lamp.

Table S4. Emission wavelength (λ_{em}) and fluorescence quantum yield (Φ) of **P5BB** in different solvents ($c = 1.0 \times 10^{-5}$ M) with different polarity.

	Hexane	Toluene	Ether	THF
λ_{em} (nm)	422	460	473	510
Φ (%)	33	24	23	12

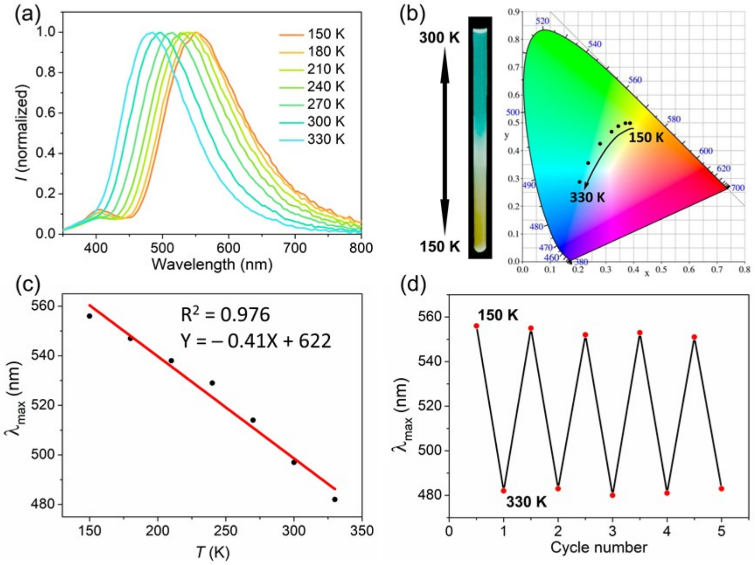

Figure S12. a) Temperature-dependent emission spectra of *rac*-**P5BB** recorded in 2-methyltetrahydrofuran between 150 and 330 K ($c = 2.0 \times 10^{-5}$ M, λ_{ex} = 306 nm); b) Photograph shown in an NMR tube and CIE color coordinates; (c) Data fitting of the emission (λ_{max}) dependence on temperature (T) for *rac*-**P5BB**, and d) reversible emission modulation with cycling T changes for *rac*-**P5BB**.

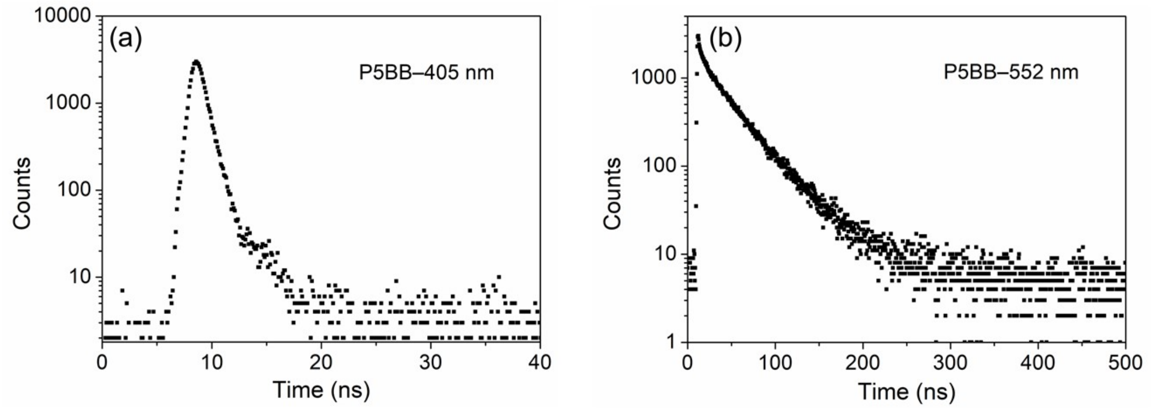

Figure S13. Transient photoluminescence decay spectra for different emission bands of **P5BB** in 2-methyltetrahydrofuran ($c = 2.0 \times 10^{-5}$ M) at 150 K.

6. DFT and TD-DFT Computations

DFT and TD-DFT calculations were performed using the Gaussian 09 suite of programs.[S4] Geometry optimizations and vertical excitations were obtained at the (DFT)B3LYP/6-31G(d,p) and (TD-DFT)B3LYP/6-31G(d) level of theory,[S5,S6] and the resulting structures were confirmed to be stationary points through vibrational frequency analysis.

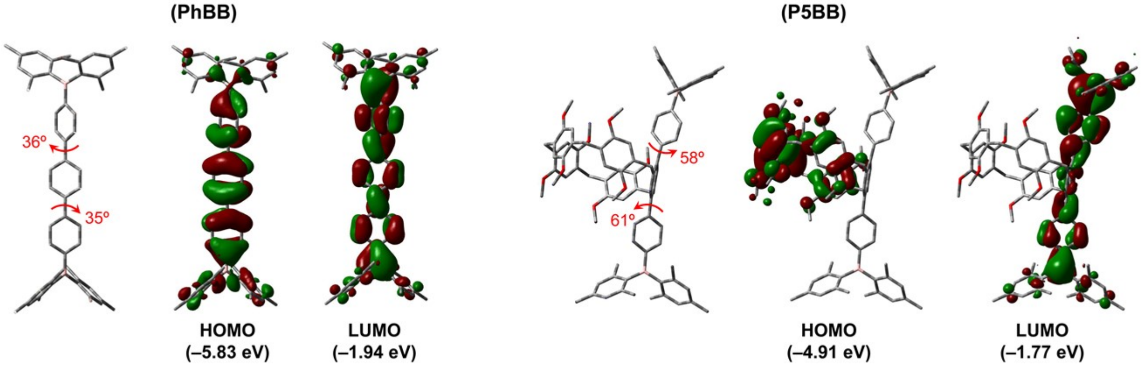

Figure S14. HOMO and LUMO distributions in **PhBB** (left) and **P5BB** (right), calculated with B3LYP/6-31G(d,p) method (iso = 0.02).

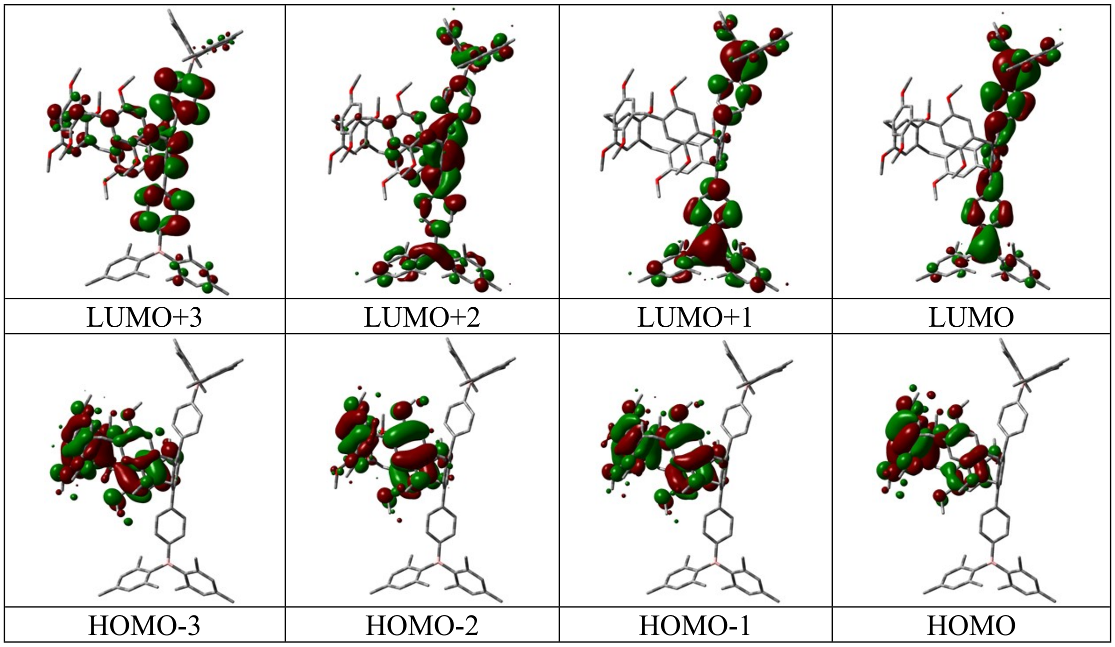

Figure S15. Molecular orbitals contributing to the DFT calculated transitions of **P5BB** (B3LYP/6-31G(p,d), iso = 0.02).

Table S5. Comparison of the TD-DFT calculations (B3LYP, 6-31G(d)) for **PhBB** and **P5BB**.

Compound	Transition	λ, nm (eV)	Oscillator Strength, *f*	Orbital Contributions
**PhBB**	$S_0 \rightarrow S_1$	367 (3.38)	1.2994	HOMO→LUMO (87%)
	$S_0 \rightarrow S_2$	361 (3.43)	0.0706	HOMO-1→LUMO (73%)<br>HOMO-1→LUMO+1 (24%)
	$S_0 \rightarrow S_3$	361 (3.44)	0.0765	HOMO-2→LUMO (73%)<br>HOMO-2→LUMO+1 (25%)
	$S_0 \rightarrow S_4$	347 (3.57)	0.0040	HOMO-4→LUMO+1 (14%)<br>HOMO-3→LUMO (71%)<br>HOMO→LUMO+1 (11%)
	$S_0 \rightarrow S_5$	342 (3.63)	0.0959	HOMO-5→LUMO+1 (10%)<br>HOMO-4→LUMO (72%)<br>HOMO-3→LUMO+1 (11%)
**P5BB**	$S_0 \rightarrow S_1$	441 (2.81)	0.0008	HOMO→LUMO (81%)
	$S_0 \rightarrow S_2$	432 (2.87)	0.0002	HOMO-1→LUMO (78%)<br>HOMO→LUMO (11%)
	$S_0 \rightarrow S_3$	418 (2.96)	0.0016	HOMO-2→LUMO (59%)<br>HOMO→LUMO+1 (27%)
	$S_0 \rightarrow S_4$	416 (2.98)	0.0001	HOMO-2→LUMO (19%)<br>HOMO→LUMO+1 (63%)

	$S_0 \rightarrow S_5$	409 (3.03)	0.0026	HOMO-1→LUMO+1 (83%)

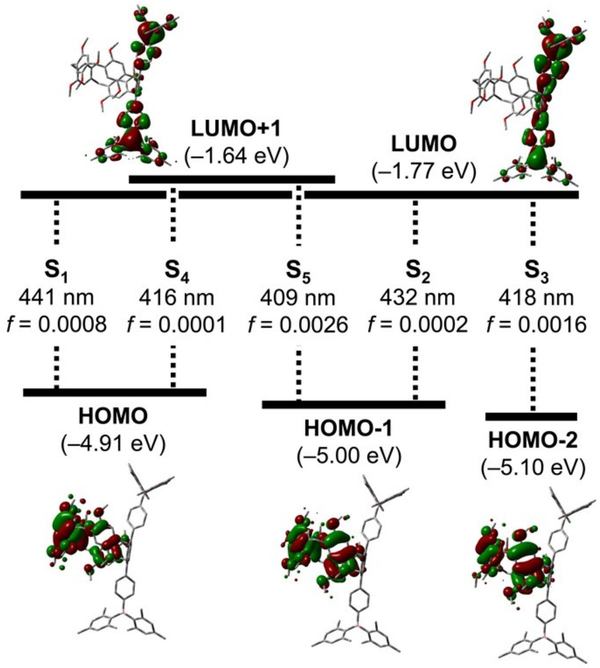

Figure S16. Key electronic transitions contributing to vertical excitations of **P5BB** (TD-DFT, B3LYP/6–31G(d)) as an example with molecular orbital plots (iso=0.02, DFT-B3LYP/6-31G(d,p)).

7. Electrochemical Measurements

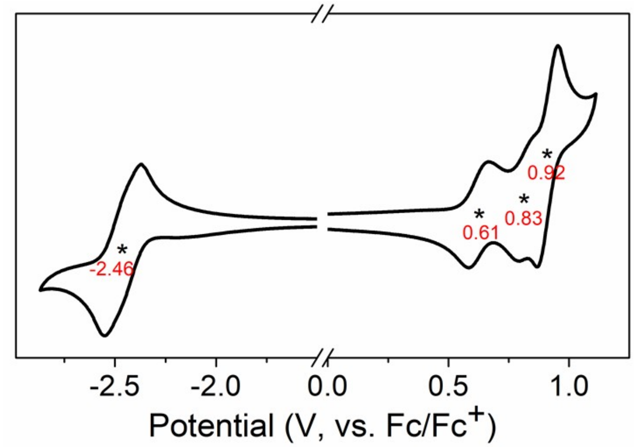

Figure S17. Cyclic voltammogram (CV) diagrams of *rac*-**P5BB** showing the reduction in THF and oxidation waves in CH_2Cl_2 *vs* Fc/Fc^+, using *n*-Bu_4NPF_6 (0.1 M) as the electrolyte, ν = 100 mV/s.

8. Chiral HPLC Trace Analysis

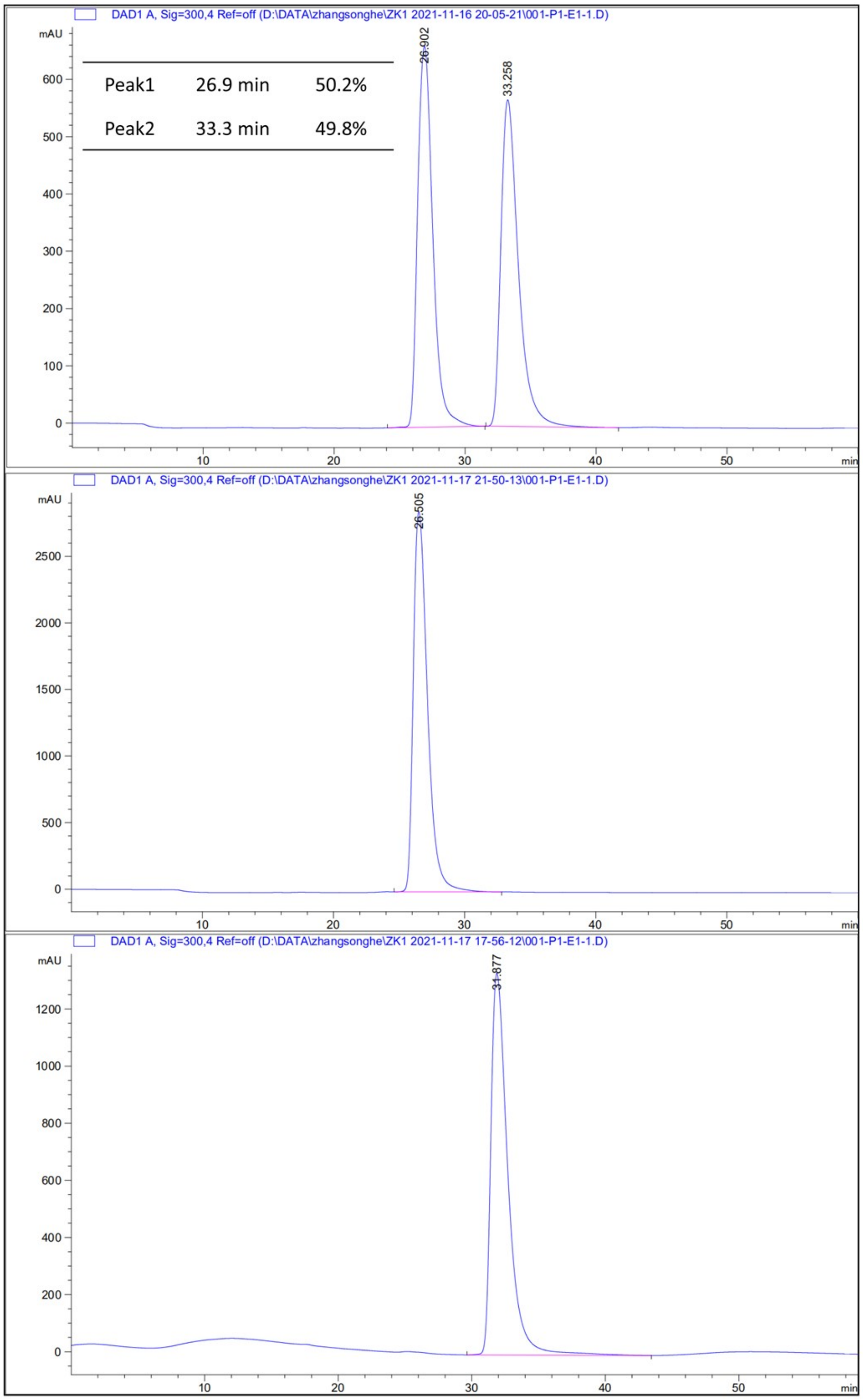

Figure S18. Chiral HPLC trace of **P5BB** (Daicel Chiralpak IB N-5 column, *n*-hexane/2-propanol = 96/4, flow rate = 1.0 mL/min, λ = 300 nm, at 30 °C).

Table S6. Summary of the HPLC analysis data of **P5BB**.

	Fraction	Retention time / min	Area / %	Enantiomeric excess (ee)	[α] / ° T = 25 °C, λ = 589 nm
rac-**P5BB**	Peak1	26.9	50.2	---	---
	Peak2	33.3	49.8	---	---
Enantiopure **P5BB**	Single Peak1	26.5	>99	>99%	−133
	Single Peak2	31.9	>99	>99%	+130

9. Host–Guest Recognition

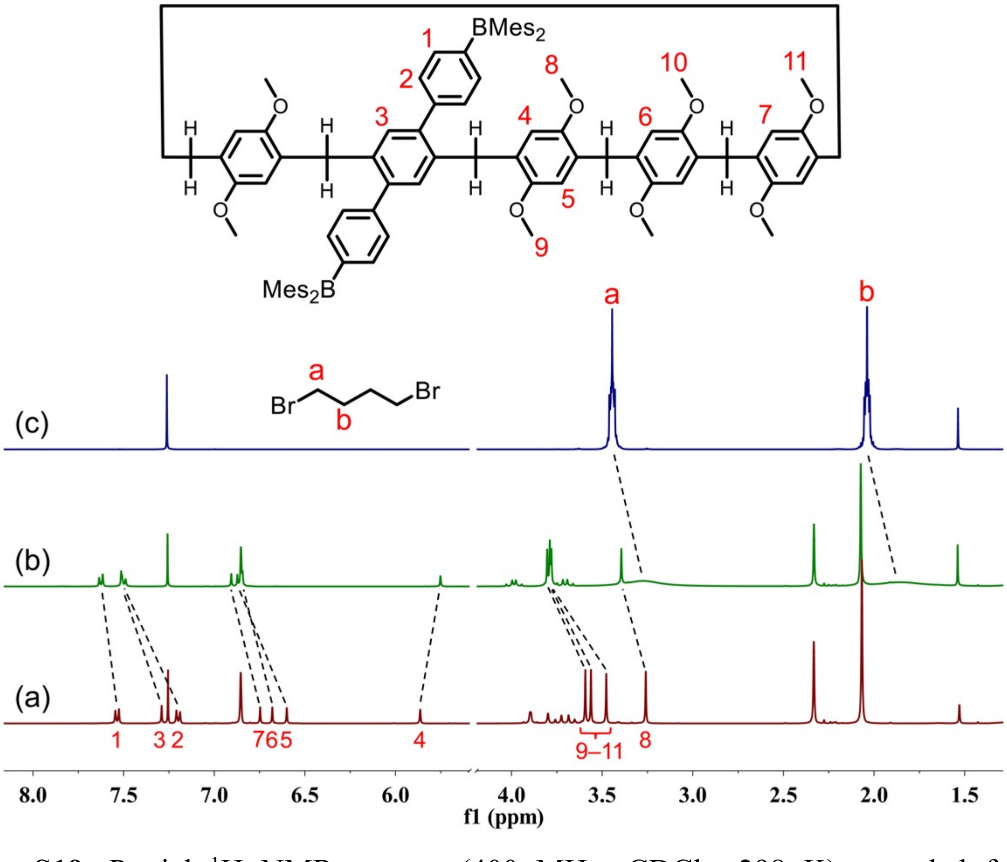

Figure S19. Partial ^{1}H NMR spectra (400 MHz, $CDCl_3$, 298 K) recorded for the host−guest recognition of **P5BB** and 1,4-dibromobutane: (a) 6 mM **P5BB**, (b) 6 mM **P5BB** + 60 mM 1,4-dibromobutane, and (c) 60 mM 1,4-dibromobutane as a neutral guest molecule.

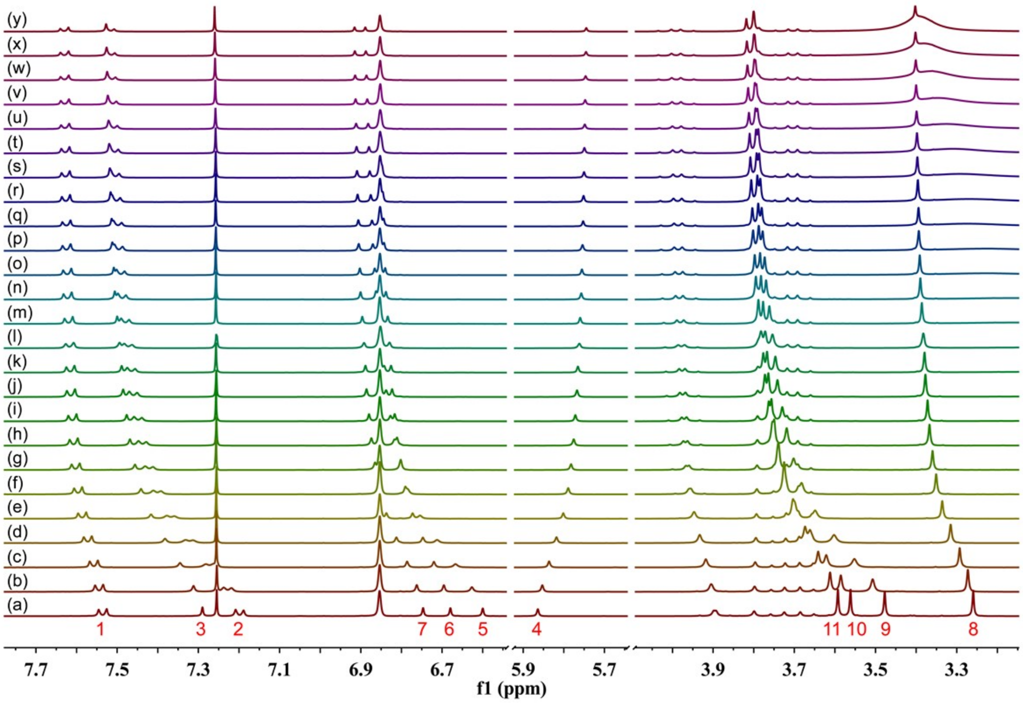

Figure S20. Partial ^{1}H NMR spectra (400 MHz, $CDCl_3$, 298 K) of **P5BB** at a concentration of 6.0 mM with different concentrations of 1,4-dibromobutane as a guest molecule: (a) 0.0 mM, (b) 1.2 mM, (c) 3.0 mM, (d) 6.0 mM, (e) 9.0 mM, (f) 12 mM, (g) 15 mM, (h) 18 mM, (i) 21 mM, (j) 24 mM, (k) 27 mM, (l)30 mM, (m) 36 mM, (n) 42 mM, (o) 48 mM, (p) 54 mM, (q) 60 mM, (r) 66 mM, (s) 75 mM, (t) 84 mM, (u) 96 mM, (v) 114 mM, (w) 138 mM, (x) 168 mM, and (y) 204 mM.

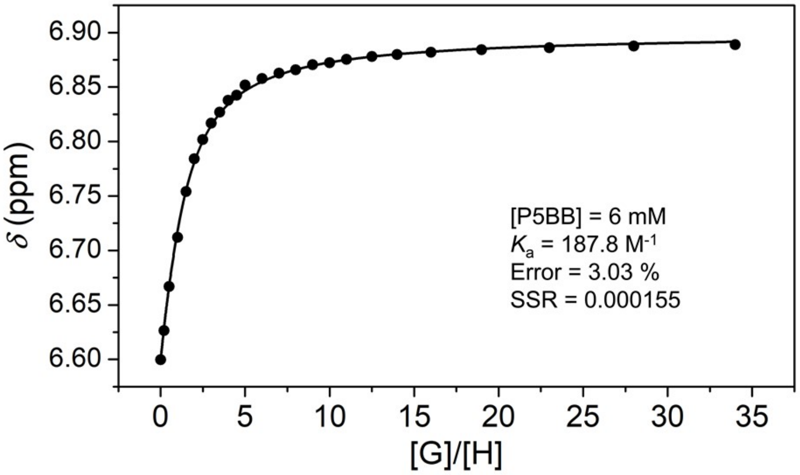

Figure S21. The chemical shift changes of H_5 on **P5BB** upon addition of different equiv. of 1,4-dibromobutane. The black solid line was fitted using Bindfit software.[S7]

10. Anion Recognition

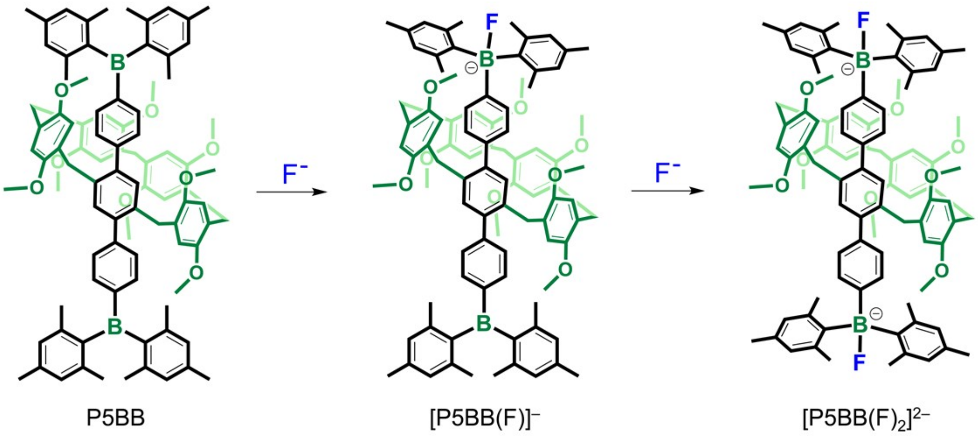

Scheme S1. Chemical structure of **P5BB** and the corresponding fluoride ion complexes.

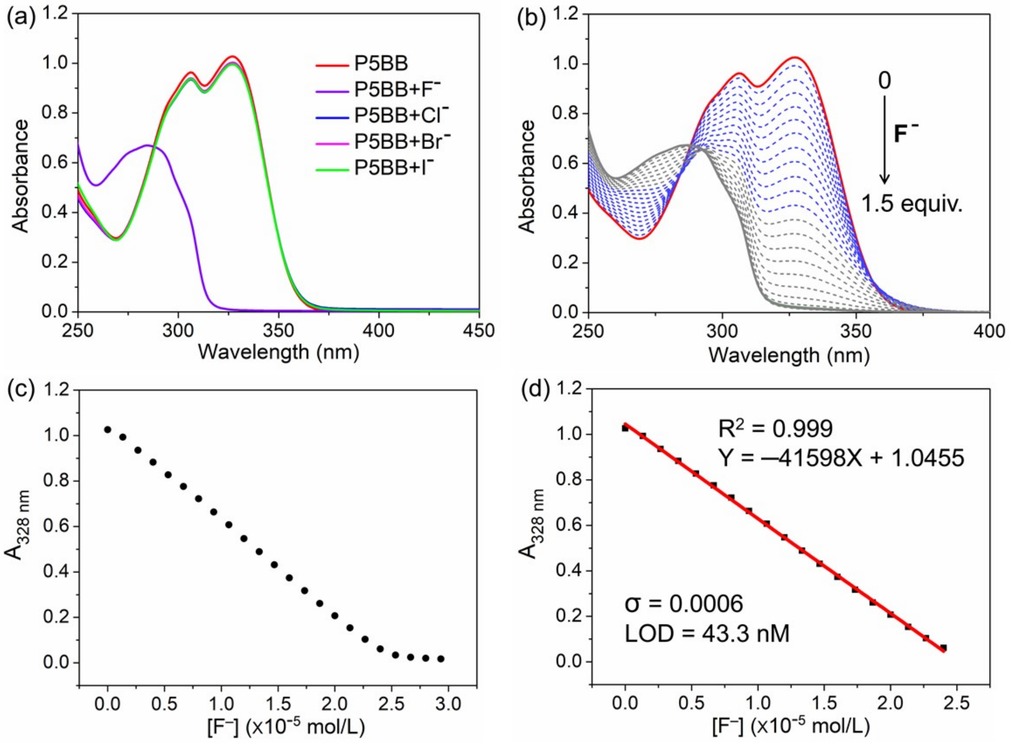

Figure S22. (a) UV−vis absorption of *rac*-**P5BB** (2.0×10^{-5} M) to various solutions of anions (5.0×10^{-5} M) as the tetrabutylammonium salts, in THF solution; (b) UV−vis absorption spectra with the addition of different concentrations of F^- to *rac*-**P5BB** in THF solution; (c) Plot of absorption at 328 nm *versus* number of equivalents of F^-; (d) Linear fitting of the absorption data upon addition of F^-.

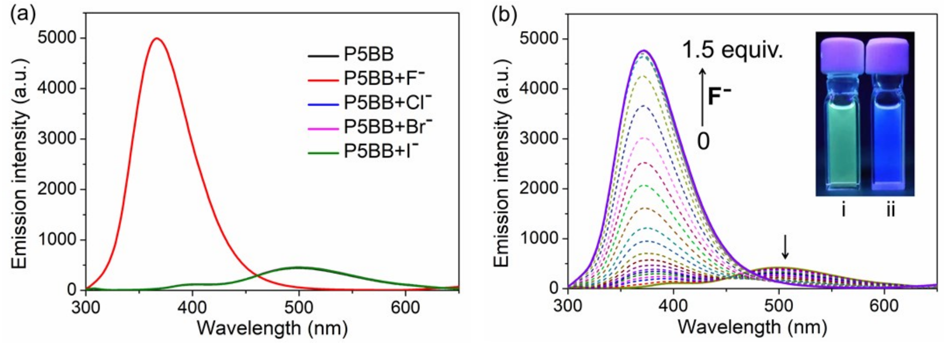

Figure S23. (a) Fluorescence responses of *rac*-**P5BB** ($c = 2.0 \times 10^{-5}$ M, $\lambda_{ex} = 306$ nm) to various solutions of anions (5.0×10^{-5} M) as the tetrabutylammonium salts, in THF solution; (b) Fluorescence spectra with the addition of different concentrations of F^- to *rac*-**P5BB** in THF solution. Inset: photographs of solutions before (i) and after (ii) the addition of F^- under 365 nm UV irradiation.

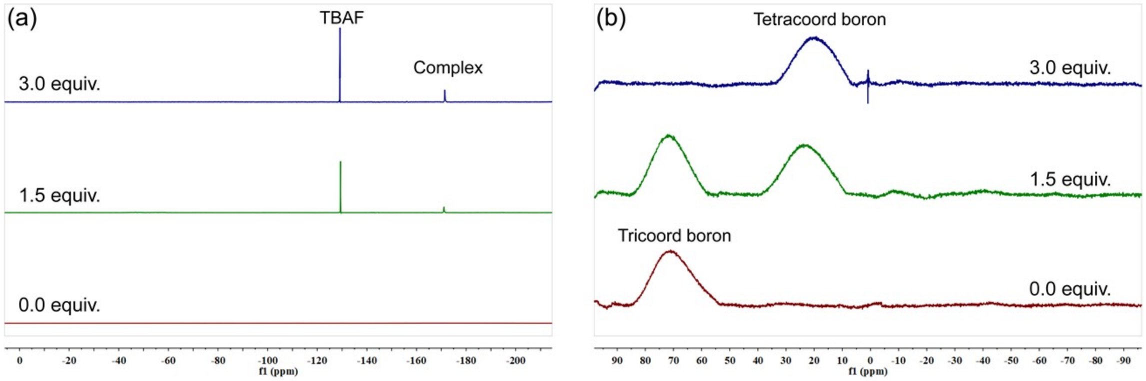

Figure S24. (a) ^{19}F NMR and (b) ^{11}B NMR spectroscopy stacked plot of **P5BB** in the presence different equivalent F^- in $CDCl_3$.

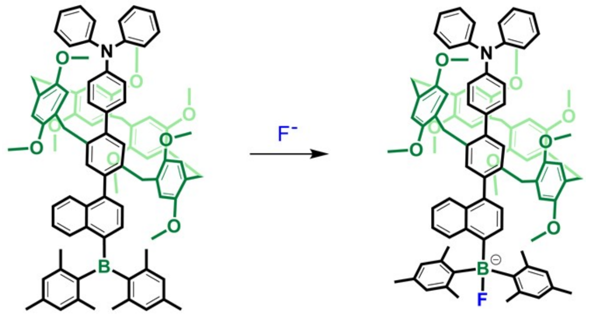

Scheme S2. Chemical structure of chiral pillar[5]arene derivative with a 1,4-naphthylene spacer and the corresponding fluoride ion complexes.

11. Coordinates (Å) for the Optimized Structures

Table S7. Coordinates (Å) for the optimized structure (B3LYP, 6-31G(d,p)) of **PhBB**.

atom	X	Y	Z	atom	X	Y	Z
C	-5.020903	1.185570	0.191845	H	-5.552600	2.118272	0.359994
C	-3.630415	1.192243	0.178753	H	-3.095944	2.122417	0.348051
C	-2.904345	0.001383	-0.005403	H	-3.096814	-2.119541	-0.359032
C	-3.630895	-1.189214	-0.189343	H	-5.553501	-2.114734	-0.368681
C	-5.021387	-1.182156	-0.201168	H	-10.418383	-3.619353	-0.311682
C	-5.763330	0.001774	-0.004110	H	-8.363104	-2.530302	-3.902720
B	-7.332425	0.001672	-0.001803	H	-8.350183	2.531997	3.903283
C	-1.422261	0.001030	-0.005152	H	-10.413502	3.626035	0.318175
C	-8.096263	1.148035	0.782192	H	-11.206189	-4.446642	-2.584126
C	-8.098268	-1.145815	-0.782300	H	-9.670315	-5.289887	-2.778599
C	-9.051237	-1.975638	-0.129446	H	-10.199882	-4.141793	-4.010160
C	-9.700810	-2.991390	-0.836044	H	-9.868627	4.367659	3.988449
C	-9.467543	-3.216567	-2.195113	H	-11.280642	4.122647	2.951869
C	-8.547710	-2.389397	-2.839458	H	-10.040471	5.282520	2.483590
C	-7.854064	-1.379881	-2.161877	H	-8.495534	1.997508	-1.971979
C	-7.848217	1.380338	2.160325	H	-10.132875	2.559276	-1.643993
C	-8.540615	2.389694	2.841318	H	-9.752909	0.832517	-1.575773
C	-9.464235	3.214885	2.202017	H	-9.769550	-0.825150	1.570326
C	-9.698193	2.994292	0.841310	H	-8.498293	-1.971487	1.975677
C	-9.050408	1.980416	0.132214	H	-10.127972	-2.556006	1.649521
C	-10.175272	-4.327808	-2.932475	H	-1.225746	-1.905881	0.985498
C	-10.199086	4.302566	2.948156	H	1.226796	-1.920593	0.958649
C	-9.375695	1.831191	-1.340290	H	1.228493	1.921602	-0.966362
C	-9.379530	-1.820577	1.341849	H	-1.224047	1.907848	-0.995653
C	-0.694302	-1.070286	0.540429	H	3.098119	-0.960052	1.921252
C	0.696122	-1.069611	0.542876	H	5.554423	-1.006665	1.896620
C	1.424999	0.000499	-0.003700	H	5.557514	1.006616	-1.897991
C	0.697074	1.070813	-0.551145	H	3.101233	0.960655	-1.926305
C	-0.693366	1.072017	-0.550113	H	8.348170	-4.516931	1.122619
C	2.907444	0.000310	-0.002675	H	10.420815	-1.485482	3.318410
C	3.632869	-0.554069	1.067630	H	8.350691	4.516354	-1.119514
C	5.023405	-0.564871	1.057745	H	10.425935	1.484640	-3.312486
C	5.766349	-0.000064	-0.000531	H	10.188960	-5.150122	2.605264
C	5.025129	0.564946	-1.059921	H	9.658415	-4.362610	4.093103
C	3.634601	0.554515	-1.071889	H	11.196331	-3.905026	3.362860
B	7.335814	-0.000213	0.000674	H	10.192860	5.149529	-2.600036
C	8.098940	-1.114741	0.829468	H	9.665587	4.361171	-4.088603
C	8.100371	1.114201	-0.826943	H	11.202157	3.904410	-3.355052
C	7.847497	-2.494080	0.602552	H	6.795774	2.329626	1.311640
C	8.537830	-3.466553	1.335372	H	5.850263	3.065577	0.031087
C	9.460917	-3.130712	2.325569	H	7.149574	3.981865	0.798758
C	9.701024	-1.773404	2.554724	H	9.775388	1.207580	1.285839
C	9.055274	-0.771750	1.825041	H	10.145312	0.717672	2.945449
C	7.848862	2.493557	-0.600302	H	8.513519	1.221226	2.511329
C	8.540447	3.465954	-1.332069	H	10.149275	-0.718551	-2.940173
C	9.464870	3.130003	-2.320959	H	8.516903	-1.221862	-2.507940

C	9.705069	1.772648	-2.549846	H	9.777325	-1.208271	-1.280958
C	9.058097	0.771089	-1.821150	H	5.849613	-3.065255	-0.031860
C	10.165250	-4.193122	3.134631	H	6.797593	-2.330249	-1.311140
C	10.170529	4.192289	-3.129024	H	7.149676	-3.982498	-0.797128
C	6.862662	2.987756	0.442741	H	-5.846743	0.961225	2.894694
C	9.390641	0.668700	2.155669	H	-6.80704	-0.484268	2.640133
C	9.393674	-0.66942	-2.151292	H	-7.140207	0.557199	4.026345
C	6.862714	-2.988120	-0.441905	H	-7.149630	-0.561949	-4.031534
C	-6.861963	0.556113	2.968135	H	-5.854722	-0.950326	-2.896003
C	-6.872725	-0.553079	-2.973037	H	-6.825955	0.489531	-2.650666

Table S8. Coordinates (Å) for the optimized structure (B3LYP, 6-31G(d,p)) of **P5BB**.

atom	X	Y	Z	atom	X	Y	Z
O	-1.105628	-0.646721	3.850852	H	0.695395	2.192678	-4.691800
C	9.585727	-6.951818	3.012300	C	-1.176886	2.711838	-3.825267
H	10.641447	-6.692761	3.162694	C	-2.308253	2.221488	-3.151664
H	9.560256	-7.942501	2.547858	C	-2.404391	0.863894	-2.835682
H	9.125564	-7.028283	4.001868	H	-3.270868	0.482874	-2.307933
B	6.910586	-2.842543	-0.366485	C	1.798677	-0.048756	-4.969265
O	3.995848	1.511734	3.563888	H	2.406731	-0.936820	-5.150745
C	8.888003	-5.922452	2.156188	H	2.398815	0.693073	-4.426419
O	-0.418637	4.464495	4.391856	H	1.490358	0.380642	-5.931393
C	8.189099	-4.858788	2.727386	C	-4.468490	2.683250	-2.225838
H	8.134944	-4.781872	3.811691	H	-4.269322	2.237016	-1.242764
O	3.657260	5.843763	0.885678	H	-4.992220	1.946884	-2.848807
C	7.552452	-3.885662	1.949037	H	-5.106449	3.559894	-2.098203
O	-1.584741	6.922762	-0.011352	C	-1.032044	4.192554	-4.140963
C	6.813879	-2.785321	2.689123	H	-0.383833	4.305111	-5.013903
H	6.856509	-1.825878	2.168672	H	-2.012685	4.601624	-4.398732
H	7.239112	-2.643154	3.687297	C	-0.455698	4.991062	-2.982182
H	5.753243	-3.029803	2.814694	C	0.932103	5.097756	-2.793619
O	1.723948	4.487793	-3.742390	C	1.441140	5.793385	-1.693962
C	7.625000	-3.938979	0.531489	H	2.508647	5.860076	-1.523797
O	-3.282217	3.145377	-2.845920	C	0.597565	6.397258	-0.756518
C	8.348089	-5.016388	-0.050591	C	-0.788946	6.306154	-0.955034
O	0.685522	-0.484116	-4.209132	C	-1.297780	5.613672	-2.057012
C	8.464759	-5.189554	-1.551114	H	-2.365467	5.515226	-2.205047
H	7.490511	-5.384740	-2.014489	C	3.128775	4.608602	-3.613509
H	9.111704	-6.037192	-1.793141	H	3.558813	4.084053	-4.469043
H	8.876880	-4.299910	-2.033665	H	3.495019	4.147495	-2.686981
C	8.946388	-5.983772	0.762045	H	3.451216	5.657852	-3.637304
H	9.479872	-6.806722	0.290706	C	-2.984243	6.930447	-0.223577
C	9.888320	0.401002	-4.375235	H	-3.404943	5.916088	-0.217282
H	10.778368	-0.141908	-4.717639	H	-3.249452	7.416095	-1.171861
H	10.232863	1.352522	-3.958142	H	-3.413605	7.500949	0.602353
H	9.279904	0.617280	-5.258402	C	1.175283	7.085989	0.470057
C	9.117436	-0.402729	-3.355683	H	0.483938	7.862617	0.805578
C	9.478209	-0.391779	-2.006050	H	2.117206	7.569326	0.200411
H	10.310683	0.231578	-1.685726	C	1.421672	6.109625	1.609718
C	8.810141	-1.165431	-1.052850	C	2.666896	5.482719	1.775464

C	9.278358	-1.071014	0.384865	C	2.851887	4.544882	2.794707
H	8.522293	-0.605459	1.028551	H	3.800710	4.038044	2.917789
H	10.183076	-0.461288	0.458591	C	1.815168	4.197133	3.665320
H	9.496703	-2.054764	0.807657	C	0.572661	4.831712	3.506137
C	7.705814	-1.976410	-1.433181	C	0.390104	5.775827	2.492327
C	7.328828	-1.976044	-2.802519	H	-0.569918	6.255051	2.345163
C	6.172500	-2.802240	-3.336765	C	4.949454	5.295060	1.069788
H	6.005592	-3.719558	-2.769049	H	5.360958	5.545656	2.056314
H	6.352929	-3.083038	-4.379011	H	5.580286	5.737809	0.296522
H	5.231999	-2.240645	-3.305405	H	4.950765	4.203177	0.954113
C	8.042080	-1.208057	-3.730519	C	-1.665371	5.130038	4.307777
H	7.746699	-1.243168	-4.777611	H	-1.559237	6.213138	4.452868
C	5.367226	-2.612177	-0.209538	H	-2.282683	4.721210	5.110146
C	4.521381	-3.608309	0.324587	H	-2.160726	4.950013	3.344570
H	4.957371	-4.547908	0.653174	C	2.023323	3.127293	4.725840
C	3.143254	-3.433716	0.411892	H	1.370821	3.333737	5.577576
H	2.517975	-4.239921	0.785219	H	3.057080	3.170519	5.077693
C	2.539272	-2.236909	-0.011953	C	1.732383	1.727821	4.206584
C	3.368896	-1.226205	-0.527899	C	2.737867	0.951635	3.608259
H	2.919906	-0.291822	-0.852044	C	2.438414	-0.315797	3.099830
C	4.744607	-1.416533	-0.630071	H	3.210824	-0.913984	2.632326
H	5.358518	-0.623857	-1.049835	C	1.145465	-0.844653	3.161903
C	1.055543	-2.076540	-0.007239	C	0.145028	-0.075330	3.779202
C	0.259434	-2.141807	1.157528	C	0.444567	1.191151	4.288699
C	-1.127093	-2.085051	0.999573	H	-0.325023	1.801634	4.744413
H	-1.745955	-2.158084	1.887283	C	5.060096	0.723829	3.059221
C	-1.758813	-1.945570	-0.246685	H	5.958811	1.337520	3.146884
C	-0.959221	-1.804672	-1.400535	H	4.907244	0.453777	2.006611
C	0.428778	-1.883871	-1.245487	H	5.195196	-0.195524	3.643536
H	1.047176	-1.799360	-2.133308	C	-2.129655	0.066762	4.520603
C	-3.249722	-1.984239	-0.263404	H	-1.870966	0.259850	5.569950
C	-3.949851	-2.954385	-1.003138	H	-3.016687	-0.568045	4.480693
H	-3.392842	-3.663782	-1.608548	H	-2.348950	1.023549	4.029010
C	-5.335976	-3.047545	-0.926854	C	0.839516	-2.212805	2.568240
H	-5.844924	-3.828248	-1.485754	H	0.123146	-2.729366	3.213305
C	-6.100917	-2.166315	-0.132957	H	1.759429	-2.801230	2.570792
C	-5.386595	-1.182637	0.583896	C	-6.872920	-4.833256	1.495510
H	-5.937911	-0.478979	1.202474	H	-5.869083	-4.984162	1.083368
C	-3.997133	-1.100940	0.535715	H	-6.848330	-3.885733	2.037641
H	-3.472678	-0.346914	1.116020	H	-7.051328	-5.629758	2.224310
C	-8.327480	-3.708327	-0.315466	C	-9.819937	-2.700510	-2.148789
C	-7.929763	-4.866117	0.406003	H	-10.136204	-1.852591	-1.536537
C	-8.533414	-6.101575	0.145219	H	-9.034339	-2.337718	-2.822580
H	-8.230411	-6.967072	0.731437	H	-10.664810	-3.001164	-2.774461
C	-9.510324	-6.256385	-0.838486	C	-10.118591	-7.606143	-1.134188
C	-9.895457	-5.119470	-1.552688	H	-9.593829	-8.105012	-1.958862
H	-10.660576	-5.211205	-2.320992	H	-10.063683	-8.269429	-0.265949
C	-9.337960	-3.862034	-1.304237	H	-11.169038	-7.516631	-1.428264
C	-8.512603	-1.027193	0.394554	C	-7.588968	0.300446	-1.607656
C	-9.360789	-1.042138	1.534624	H	-7.934994	1.131281	-2.229790
C	-10.087694	0.099553	1.886794	H	-6.541361	0.488575	-1.350090

H	-10.723811	0.064294	2.768959	H	-7.600258	-0.601558	-2.224691
C	-10.033420	1.275272	1.135600	C	-9.488805	-2.261378	2.424554
C	-9.214300	1.283438	0.005510	H	-9.783607	-3.149045	1.859425
H	-9.170212	2.179993	-0.609993	H	-8.543094	-2.499376	2.925560
C	-8.449525	0.172031	-0.364540	H	-10.234371	-2.093340	3.206584
C	-1.520258	-1.508626	-2.789203	C	-10.819172	2.498648	1.542446
H	-0.988064	-2.117128	-3.525467	H	-10.255826	3.113092	2.256345
H	-2.573925	-1.787729	-2.837949	H	-11.049529	3.132018	0.680559
C	-1.394776	-0.041889	-3.176451	H	-11.762423	2.226381	2.025950
C	-0.274749	0.444567	-3.868877	B	-7.659780	-2.299480	-0.021842
C	-0.176521	1.803996	-4.181169				

References:

[S1] X. Li, Z. Li, Y.-W. Yang, *Adv. Mater.* **2018**, *30*, 1800177.

[S2] X. Xu, S. Ye, B. He, B. Chen, J. Xiang, J. Zhou, P. Lu, Z. Zhao, H. Qiu, *Dyes Pigm.* **2014**, *101*, 136–141.

[S3] Y.-J. Na, W. Song, J. Y. Lee, S.-H. Hwang, *Dalton Trans.* **2015**, *44*, 8360–8363.

[S4] M. J. Frisch, G. W. Trucks, H. B. Schlegel, G. E. Scuseria, M. A. Robb, J. R. Cheeseman, G. Scalmani, V. Barone, B. Mennucci, G. A. H. Petersson, M. Nakatsuji, X. Caricato, H. P. F. Li, A. Hratchian, J. Izmaylov, G. Bloino, J. L. Zheng, M. Sonnenberg, M. Hada, K. Ehara, R. Toyota, J. Fukuda, M. Hasegawa, T. Ishida, Y. Nakajima, O. Honda, H. Kitao, T. Nakai, J. A. Vreven, J. E. Montgomery Jr., F. Peralta, M. Ogliaro, J. J. Bearpark, E. Heyd, K. N. Brothers, V. N. Kudin, T. Staroverov, R. Keith, J. Kobayashi, K. Normand, A. Raghavachari, J. C. Rendell, S. S. Burant, J. Iyengar, M. Tomasi, N. Cossi, J. M. Rega, M. Millam, J. E. Klene, J. B. Knox, V. Cross, C. Bakken, J. Adamo, R. Jaramillo, R. E. Gomperts, O. Stratmann, A. J. Yazyev, R. Austin, C. Cammi, J. W. Pomelli, R. L. Ochterski, K. Martin, V. G. Morokuma, G. A. Zakrzewski, P. Voth, J. J. Salvador, S. Dannenberg, A. D. Dapprich, O. Daniels, J. B. Farkas, J. V. Foresman, J. Ortiz, J. Cioslowski, D. J. Fox, Gaussian 09 Revision C.01, 2010.

[S5] (a) C. Lee, W. Yang, R. G. Parr, *Phys. Rev. B*, **1988**, *37*, 785–789; (b) A. D. Becke, *J. Chem. Phys.* **1993**, *98*, 1372–1377; (c) A. D. Becke, *J. Chem. Phys.* **1993**, *98*, 5648–5652.

[S6] (a) R. Ditchfield, W. J. Hehre, J. A. Pople, *J. Chem. Phys.* **1971**, *54*, 724–728; (b) A. D. McLean, G. S. Chandler, *J. Chem. Phys.* **1980**, *72*, 5639–5648.

[S7] The binding constant *K* is determined with the online tools at http://supramolecular.org.
